# Extraction of Polyphenols from Slovenian Hop (*Humulus lupulus* L.) Aurora Variety Using Deep Eutectic Solvents: Choice of the Extraction Method vs. Structure of the Solvent

**DOI:** 10.3390/plants12162890

**Published:** 2023-08-08

**Authors:** Ilir Metaj, Drilon Hajdini, Kaja Gliha, Iztok Jože Košir, Miha Ocvirk, Mitja Kolar, Janez Cerar

**Affiliations:** 1 Department of Food Technology with Biotechnology, Faculty of Agriculture and Veterinary, University of Prishtina “Hasan Prishtina”, 10000 Prishtina, Kosovo; ilir.metaj1@student.uni-pr.edu (I.M.); driloni099@gmail.com (D.H.); 2 Faculty of Chemistry and Chemical Technology, University of Ljubljana, Večna Pot 113, 1000 Ljubljana, Slovenia; kg5120@student.uni-lj.si (K.G.); mitja.kolar@fkkt.uni-lj.si (M.K.); 3 Department for Agrochemistry and Brewing, Slovenian Institute of Hop Research and Brewing, 3310 Zalec, Slovenia; iztok.kosir@ihps.si (I.J.K.); miha.ocvirk@ihps.si (M.O.)

**Keywords:** ultrasound-assisted extraction, density, viscosity, speed of sound, adiabatic compressibility, hydrophobicity

## Abstract

Polyphenols from Slovenian hops (*Humulus lupulus* L.) of the Aurora variety were extracted by different methods and using classical solvents and several deep eutectic solvents (DES) based on choline chloride as the hydrogen bond acceptor component. The obtained extract solutions were analyzed by HPLC for the content of extracted α- and β-acids and extracted xanthohumol. It was found that choline chloride:phenol DES concentrated aqueous solution had an extraction efficiency close to that of diethyl ether, which is considered one of the best classical extraction solvents for polyphenols from hops. The comparison of the extraction efficiency with other choline chloride-based DESs showed that the chemical similarity of the phenol ring in the solvent DES with the polyphenols in hops may be crucial for a highly efficient extraction with choline chloride:phenol DES. On the other hand, the choice of extraction method and the viscosity of the solvents tested seem to play only a minor role in this respect. As far as we know, this is the first study to attempt to relate extraction efficiency in the extraction of hydrophobic solutes to the compressibility of the DES extractants, the latter of which may be correlated with the extent of hydrophobic hydration around the DES components. In addition, using the heating and stirring method for the preparation of choline chloride-based DES concentrated aqueous solutions we found no support for the occurrence of water in two different roles (in the structural and in the dilution role) in these solvents.

## 1. Introduction

Deep eutectic solvents (DESs) are typically described as mixtures of two or more solid organic or inorganic compounds that form a stable eutectic under optimal conditions [1]. At first glance, the properties of DESs resemble those of ionic liquids (ILs), and one could even call them their analogues [2,3]. A closer look reveals that the physical properties of DESs are indeed similar to those of ILs, while their chemical properties are so different that the applications of DESs and ILs differ considerably. This is a consequence of the fact that ILs are composed exclusively of discrete cations and anions, whereas DESs are formed from Lewis or Brønsted acids and bases, which may (but need not) be composed of various anionic and/or cationic species [2]. Applications (or at least suggestions for them) of DESs include areas such as metal processing applications (metal electrodeposition, metal electropolishing, metal extraction), separations, gas capture, power systems, battery technologies, biocatalysis, and biomass processing, to name only the most well-known ones [2,3].

In addition to being non-toxic or what is now referred to as “green”, DESs improve extraction efficiency, increase the stability of extracts, and offer a whole new perspective in the isolation of bioactive compounds [4]. The structure of eutectics is usually explained by the formation of hydrogen and Van der Waals bonds, but this issue is still debated in the scientific community [5,6]. Since the publication of Abbott et al. in which the use of choline chloride was first described, this compound has remained one of the most popular starting materials for DESs preparation due to its low melting point, water solubility, low price, and accessibility [7]. Choline chloride (ChCl) is usually chosen as a hydrogen bond acceptor (HBA); however, it also functions as a quaternary ammonium salt, while the typical hydrogen bond donors (HBDs) are sugars, urea, polyalcohols, organic acids, or phenols [8].

The physical and chemical properties of DESs depend strongly on the chemical properties of the acceptor and donor compound, their molar ratio, temperature, and water content [9]. For practical applications, one needs to consider the density, viscosity, polarity, surface tension, and pH values when DES solvents are used. In general, DESs have a higher density than water, with experimentally reported values up to 1.6 g/mL, but this can be regulated with water addition, typically in the range of 5 and up to 25% *w*/*w*. On the other hand, strong intermolecular interactions among DES components that are responsible for low vapor pressure and high viscosity of DES-based solvents have both positive and negative aspects when extractions are considered. The positive aspect is that the temperature of the system In question can be increased without losing the extraction solvents, while the negative aspect is that the DES-based solvent is very difficult to remove when dry extracts are required or in industrial scale-ups.

Due to all these issues, the physicochemical properties of DESs are frequently studied [10,11,12]. Studies of these kinds are usually focused on exploring the influence of DES composition on the melting point, densities, viscosities, electric conductivities, surface tension, refractive index, pH-value, and hydrophobicity (see e.g., [7,13,14,15,16,17,18,19])—sometimes being focused only on one such property but very often on several of them. In addition, considering the negative impact of a high viscosity of DESs on extraction efficiency, it is not surprising that DESs are often prepared in the form of concentrated aqueous DES solutions and that the influence of water on physicochemical properties of such systems is therefore frequently studied [20,21]. Moreover, it was recently reported that not only the water content but also the stage of preparation of concentrated DESs in which water is added to DES influences the physicochemical properties of aqueous DES solutions [22].

Yet, it is not only solvents that may be crucial for successful extraction. Considering active compounds from plants, the classical extraction methods are often criticized for not being efficient enough when compared to more advanced techniques [23]. Nevertheless, high temperatures may lead to the degradation of extracted molecules which also limits the assortment of extraction methods used, e.g., for the extraction of phenolic compounds [24,25]. Further—as one of the recent studies has shown—activity of extracted bioactive components may depend on the choice of the selected extraction method [26].

The majority of the above mentioned and many other aspects of DESs were recently addressed by our group in the paper entitled: Innovative extraction techniques using deep eutectic solvents and analytical methods for the isolation and characterization of natural bioactive compounds from plant material [27]. In addition to DES properties and selection, the isolation of bioactive compounds from plants and various analytical methods were presented and discussed. We were especially focused on plants: *Achillea millefolium* L. [28], *Helichrysum arenarium* L. [29], olive leaves [30], *Hibiscus sabdariffa* L. [31], *Aronia melanocarpa* [32], *Coriandrum sativum* L. [33], and *Lippia citriodora* [34]. In general, bioactive compounds were successfully isolated for all listed plants by using selected and optimized deep eutectic solvents.

In this work, we continue with exploiting the possibilities offered by DESs for the efficient extraction of biologically active compounds from plants, focusing now on hops as an important industrial plant for the beer industry. Although numerous papers have been published on the use of DESs in the extraction of bioactive compounds from plants, both of the research and of the review type [35,36,37,38,39,40,41], there is a lack of general or specific information on how to address the problem of the extraction of major phenolic compounds (α- and β-acids, xanthohumol) from hops. While there are papers reporting the extraction of phenolic acids and flavonoids from plants using DESs [42], these conclusions may not be directly applicable to α- and β-acids, as it can be assumed that these acids are less polar and more hydrophobic than the most commonly studied phenolic acids. Similarly, xanthohumol, which belongs to prenylated flavonoids (more specifically, prenylated chalcones), may be considered more hydrophobic than normal flavonoids, as indicated by the adjective “prenylated”. Nevertheless, the reports of other researchers on the extraction of similar phenolic compounds from plants using DESs may be a good starting point for the design of our study.

For example, Gao et al. attempted to extract seven major phenolics (four of which are acids and three flavonoids) from mulberry (*Morus alba* L.) leaves. This was most efficiently achieved with ChCl-glycerol DES in a molar ratio of 1:2 with the addition of 20% water using the DES-MAE (microwave-assisted extraction) method [43]. Similarly, attempts were made to extract caffeic acid and some other phenolic compounds from olive pomace using different ChCl-based DESs [44]. The best results were obtained with solvents prepared from ChCl-citric acid or ChCl-lactic acid DESs with a molar ratio of 1:2 and 20% (*v*/*v*) water using the DES-HAE (homogenizer-assisted extraction) method. These combinations were more effective than using the conventional solvents (70% *v*/*v* aqueous ethanol or pure water). Thirty-one similar studies reporting the extraction of phenolic compounds from plants are further mentioned in the review by Redha [45]. By far the most common HBA component in the DESs used in these studies was ChCl, while a small number of studies (also) included L-proline, betaine, betaine hydrochloride, glycine, L-alanine, nicotinamide, citric acid, or salts (ammonium acetate, sodium acetate, sodium potassium tartrate). For HBD components, a wider range of compounds was used. The most typical were glycerol, ethylene glycol, urea, glucose, lactic acid, tartaric acid, oxalic acid, and citric acid. In addition, many other HBD compounds less commonly used in the preparation of DESs were also used in these studies; in two papers, the authors used HBDs containing an aromatic ring. In the first of these two studies, phenyl-based acids were used in combination with ChCl as the HBA component for the extraction of the phenolic compounds from walnut (*Juglans regia* L.) leaves. However, the presence of the aromatic ring in the HBD component made no difference when compared to the use of organic acids without the π-system, and the extraction efficiency remained of the same order of magnitude [46]. In contrast, in the second of these two studies authored by Ali et al. [47], it was found that the replacement of HBD components such as 1,2-propanediol, glycerol, ethylene glycol, malic acid, levulinic acid, xylitol, and urea with *p*-toluenesulfonic acid in DESs in which ChCl was used as the HBA component, significantly (almost by an order of magnitude compared to the second most effective DES extractant) increased the extraction yield in the extraction of myricetin and rutin from matrimony vine (*Lycium barbarum* L.) fruits. The same extractant was also at least as efficient as other DES extractants used in the extraction of the other phenolics monitored (morin, luteolin, hyperoside, quercitrin, apigenin). However, in the same study, resorcinol (IUPAC name: benzene-1,3-diol) was also used as an alternative HBD component with an aromatic ring, and the extraction efficiency of DESs in this case was similar to that of the non-aromatic HBD components. It should be added that in this study by Ali et al., the addition of water to DES solvents decreased the extraction efficiency.

Few data are available on the use of DESs for the extraction of phenolics from hops. The study conducted by Lakka et al. focused on the optimization and kinetics of the extraction of polyphenols from hops using L-alanine-glycerol DES (70% *w*/*w* aqueous mixture) using an ultrasound-assisted pretreatment [48]. The comparison of extraction efficiencies for total polyphenols and total flavanols showed that L-alanine-glycerol DES was similarly efficient as a 60% (*v*/*v*) aqueous solution of methanol or ethanol in extracting these compounds and slightly less efficient in extracting total flavonoids. For all three groups studied (total polyphenols, total flavanols, total flavonoids), extraction with DES was more efficient than extraction with pure water. In the two studies by Grudniewska et al. [49,50], four choline chloride-based DESs with glycerol, ethylene glycol, propylene glycol, or lactic acid as the HBD component (with the addition of 5–10% *w*/*w* of water) were used for the extraction of xanthohumol from spent hops. All four DES solvents were similarly effective in extraction, and no comparison was made to the extraction with classical solvents. As far as we are aware, the list of published research papers in which DESs were used for the extraction of α- or β-acids or xanthohumol from hops ends with the study of Macchioni et al. [51], in which three lactic acid (LA)-based DESs were used to investigate the extraction efficiency of these DESs for the extraction of α- and β-acids as well as non-phenolic pigments and polyphenols. Sucrose, urea, and glycine were used as HBD components in the preparation of the three DES solvents mentioned above. The most successful extraction was observed with LA-sucrose (molar ratio 4:1), followed by LA-urea (3:1), and finally LA-glycine (3:1) DES. All these extractants contained 20% (*w*/*w*) water. The extraction efficiency was compared with the extraction using 80% acidified methanol. All the extractants used were more successful than the control solvent in the extraction of α-acids, while in the extraction of β-acids, the performance of LA-glycine DES was worse than that of the control solvent.

It is our intention to broaden this kind of knowledge. In our case, it is a comparison of the extraction efficiency of α- and β-acids, as well as of xanthohumol from hops (*Humulus lupulus* L.) The aurora variety was studied for several standard organic solvents and several DES-based solvents, using various extraction methods. A premise is made, that using choline chloride as the HBA and a suitable HBD molecule being chemically similar to extracted phenolic compounds, it is possible to prepare DES that has a similar extraction efficiency as the best organic solvents used for the extraction of α- and β-acids, as well as xanthohumol from hop. We expect that the choice of the extraction method—having in mind limits given by the properties of extraction solvents and the extracted compounds—is of lesser importance than the chemical similarity of the DES used.

In order to further contribute to a better understanding of DESs, the prepared concentrated aqueous solutions of DESs used in the extraction were physicochemically characterized by measuring their densities, viscosities, and adiabatic compressibilities in the temperature range between 5 and 60 °C. In addition, for several prepared concentrated aqueous solutions of DESs it was shown that the time of adding water to DES does not influence their physicochemical properties if a heating and stirring preparation method is used.

## 2. Materials and Methods

### 2.1. Materials and Chemicals

The hops were provided by the Hmezad export import company in the shape of hop pellets type 90. In the study, the Aurora hop variety was used. This variety is the most commonly grown hop variety in Slovenia and is recognized for its pleasant hop aroma and bitterness that gives it an excellent brewing value. It was obtained through breeding, crossing the English variety Northern Brewer and Slovenian genetic hop germplasm [52]. Just before the beginning of the extraction procedure, the hop pellets were grinded in a grinder.

D-(−)-fructose (≥99%), D-(+)-glucose (anhydrous, 96%), urea (≥99%), 1,3-dimethylurea (98%), and hexane (≥99%) were purchased from Sigma-Aldrich (St. Louis, MO, USA). Phenol (≥99.5%), ethylene glycol (≥99%), tartaric acid (≥99.5%), toluene (≥99.5%), ethyl acetate (LiChrosolv^®^), and acetone (≥99.5%) were supplied by Merck (Germany) while lactic acid (88–92%) and glycerol (≥99.5%) were obtained from Carlo Erba (Italy). Benzenesulfonic acid (≥98%) was purchased from Fluka Chemie AG (Switzerland), diethyl ether (>99%) from J.T. Baker (USA), and choline chloride (99%) from Acros Organics (Belgium). In the preparation of concentrated aqueous DES solutions, ultrapure (type 1) water was used.

### 2.2. Preparation of the Deep Eutectic Solvents (DESs)

All together, ten different deep eutectic solvents (DESs; Table 1) were prepared following the standard heating and stirring method [53] taking into account the specific conditions already experienced with these kinds of DESs [29]. In order to reduce the viscosity of DESs and thus facilitate the extraction, we decided at the beginning of the study to use DESs containing 25% (*w*/*w*) of water. With the aim of simplifying the preparation of these concentrated aqueous DES solutions, the original heating and stirring method [53] was modified. In this modified preparation procedure already at the beginning of DES preparation (i.e., before heating and stirring were started), the calculated amount of ultrapure water corresponding to 1/3 of the mass of DES components was added to the dispensed DES components. Consequently, the final concentrated aqueous DES solutions contained 75% DES and 25% water (*w*/*w*%). To achieve this goal, each component of DES as well as the above mentioned extra added water were put together in a closed glass flask and then immersed in a water bath at a temperature of 80 °C. This mixture was heated and stirred at 500–600 RPM until a clear solution was formed (a time scale of one hour). After the preparation, the prepared solvents were put in closed plastic tubes and stored at room temperature.

In order to verify the equivalency of this preparation procedure with the established one (first anhydrous DES is prepared and then water is added to the anhydrous DES), four out of a total ten prepared concentrated aqueous DES solutions were prepared according to the original and to the modified procedure and no difference exceeding the usual variation of the physicochemical properties (density of solutions, viscosity, adiabatic compressibility) between these pairs was observed. A comparison of the properties of these concentrated aqueous DES solutions prepared by both procedures is graphically represented in the Supplementary Material (Figures S1–S4).

### 2.3. Soxhlet Extraction

An amount of 5.0 g of grinded hop pellets were put into a thimble and covered with cotton wool. The 1000 mL distillation flask was filled with 250 mL of the chosen organic solvent (methanol, acetone, ethyl acetate, hexane, or diethyl ether). Five extraction cycles were carried out with the chosen solvents. During each cycle, around 160 mL of the solution containing extracts from the hops was drained from the Soxhlet chamber. The heating power of the distillation flask was set so that the time needed for one extraction cycle was approximately 45 min.

### 2.4. Orbital Shaker Extraction (OSE)

An amount of 3.0 g of grinded hop pellets and 30 mL of solvent (either organic solvent or aqueous DES solution) was dosed into 150 mL erlenmeyer flask, closed with a stopper, and put into the thermostated (20 °C) shaker (New Brunswick Innova 4230 Refrigerated Incubator Shaker) to be shaken for 40 min at 125 RPM. After shaking, the extraction mixture was transferred into a 50 mL conical centrifuge tube and centrifuged (10,350 RPM) in the benchtop Legend Mach 1.6 R centrifuge (Thermo Scientific/Sorvall) at 20 °C for 30 min. In case of only partially successful separation of the extracted hops pellets, the supernatant containing remnants of hop pellets was transferred into unused 50 mL conical centrifuge tubes and centrifugation was repeated. While centrifugation worked well for all solutions where organic solvents were used and for the majority of DES solutions, it was not possible to obtain clear supernatant solution with ChCl-U and ChCl-DMU solutions where some haziness remained even after the second centrifugation. The supernatant phase was then transferred into a new tube, tightly closed with the screw cap, and stored in the refrigerator at 6 °C until the HPLC analysis was carried out (up to 7 days).

### 2.5. Ultrasound-Assisted Extraction Using Ultrasonic Cleaning Bath (UAE)

An amount of 0.5 g of grinded hop pellets and 5 mL of chosen solvent (either organic solvent or aqueous DES solution) was put into a 15 mL conical centrifuge tube and tightly closed with the screw cap. Then, the tube was inserted into the ultrasonic cleaning bath (Elmasonic S 40 H, Elma, Germany) for 30 min at 25 °C. Afterwards, the extraction mixture was centrifuged and stored using the procedure described in Section 2.4.

### 2.6. Ultrasonic Homogenizer Extraction (UHE)

Amounts of 1.0 g of grinded hop pellets and 10 mL of aqueous DES solution were put into a 15 mL conical centrifuge tube and sonicated for 15 min using a UP100H Ultrasonic processor (Hielscher, Germany). The amplitude was set to 50% and cycle setting to 0.6. The centrifuge tube containing the extraction mixture was kept in an ice bath during the sonication in order to prevent excessive heating of the mixture. The extraction mixture was afterwards centrifuged and stored using the procedure described in Section 2.4.

### 2.7. Chromatographic Analysis of Content of Extracted Bitter Acids and Xanthohumol

**Figure 1:**
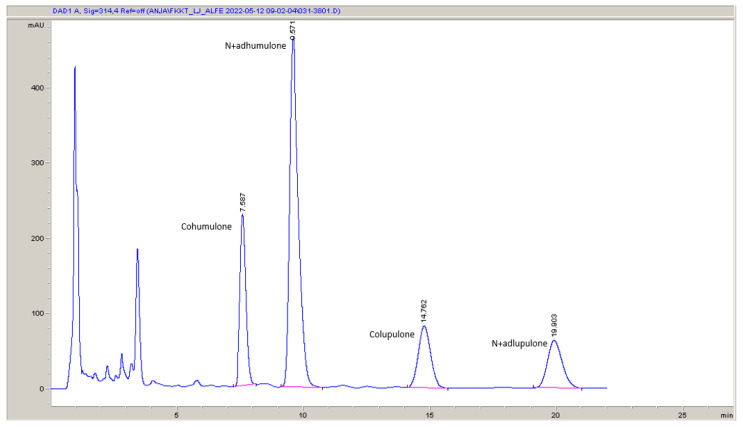
An example (sample 34—see Section 2.11) of the HPLC-DAD chromatogram used for the determination of α- and β-acids. The wavelength was 314 nm.

**Figure 2:**
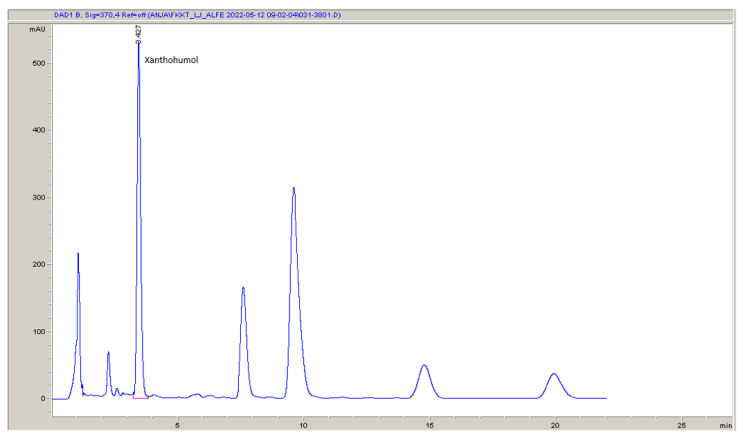
An example (sample 34—see Section 2.11) of the HPLC-DAD chromatogram used for the determination of xanthohumol. The wavelength was 370 nm.

High-performance liquid chromatography (HPLC) was used to determine α- and β-acids and xanthohumol in the hop according to the Analytica-EBC 7.7 method [54]. The separation was achieved on a Nucleodur 5–100 C18, 125 × 4 mm HPLC analysis column (Macherey-Nagel, Düren, Germany) while a 10 µL injection loop on an HPLC injector was used. The isocratic mobile phase constituted distilled water, methanol (Sigma-Aldrich, Taufkirchen, Germany), and 85% aqueous solution of ortophosphoric acid (Sigma-Aldrich, Germany) in a ratio of 775/210/9 (*v*/*v*/*v*). The detection was carried out with a diode array detector (DAD) set at 314 nm for α- and β-acids (Figure 1) and 370 nm for xanthohumol (Figure 2). The quantification was performed according to the external standard ICE4 (Labor Veritas, Zürich, Switzerland). All solvents were of analytical grade purity.

Just before the analysis, extraction solutions were volumetrically diluted by a factor of 10 with a mobile phase and filtered through disposable syringe filters, Chromafil Xtra PET-45/25 (Macherey-Nagel, Germany).

### 2.8. Measurements of Densities and Speed of Sound

The densities (*d*, kg m^−3^) of the concentrated aqueous DES solutions (DES/water, 75%:25% *w*/*w*) as well as the speed of sound propagation (*c*, m s^−1^) in these solutions were measured in the temperature range between 5 °C and 60 °C (278.15–333.15 K), in increments of 5 °C at an ambient pressure of 0.1 MPa using an Anton Paar DSA 5000 M instrument. The instrument was calibrated prior to use. According to the technical data of the measuring performance of the instrument given by the Anton Paar company, the accuracy of temperature is ±0.01 °C, the accuracy of density ±7 × 10^−6^ g/cm^3^, and the standard deviation of sound velocity is 0.5 m/s.

### 2.9. Measurements of Viscosity

Dynamic viscosities (h, mPa·s) were measured in the same temperature range as density and speed of sound (from 5 °C to 60 °C) using an Anton Paar Lovis 2000 M/ME rolling-ball viscometer. In order to obtain the optimal run times of the measurements, for these measurements the capillary with a diameter of 1.8 mm was chosen. Steel balls were used for most measurements, except for the measurement of the viscosity of the ChCl-LA, ChCl-TA, and ChCl-BSA sample, where gold-plated balls were used to avoid corrosion of the balls. Before use, the measuring system was calibrated with the viscosity standard.

### 2.10. Calculations of Adiabatic Compressibility

Adiabatic compressibilities, b_S_, were calculated from the measured speeds of sound propagation, *c*, and densities, *d*, using the established relation [55]

(1) $$\beta_{S}=\frac{1}{c^{2}d}$$

### 2.11. Legend to Figure 3, Figure 4 and Figure 5

**Figure 3:**
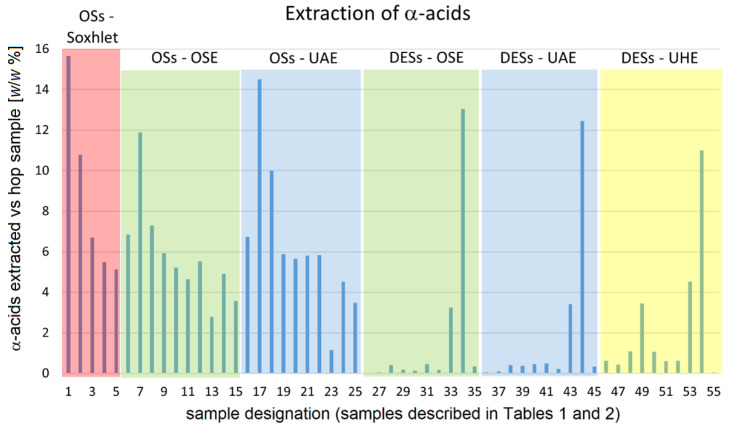
Quantity of α-acids extracted (reported as weight% of grinded hops pellets) as a function of both the extraction method and of the kind of extraction solvent used. Abbreviations: OSs-Soxhlet → organic solvents, Soxhlet extraction; OSs-OSE → organic solvents, orbital shaker extraction; OSs-UAE → organic solvents, ultrasound-assisted extraction; DESs-OSE → Deep Eutectic Solvents, orbital shaker extraction; DESs-UAE → Deep Eutectic Solvents, ultrasound-assisted extraction; DESs-UHE → Deep Eutectic Solvents, ultrasonic homogenizer extraction. Typical relative error in quantity of extracted α-acids is estimated to be around 3%.

**Figure 4:**
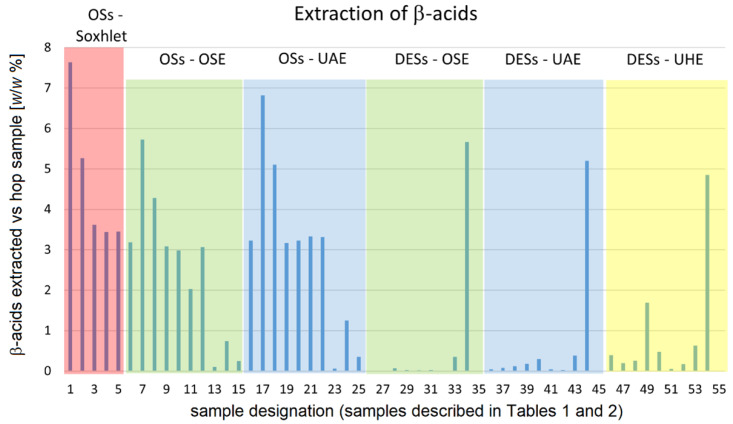
Quantity of β-acids extracted (reported as weight % of grinded hops pellets) as a function of both the extraction method and of the kind of extraction solvent used. Typical relative error in quantity of extracted β-acids is estimated to be around 3%. The abbreviations are explained in the caption to Figure 3.

**Figure 5:**
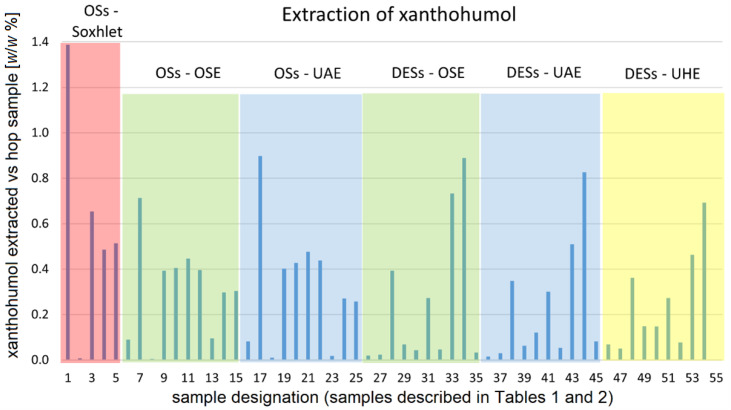
Quantity of xanthohumol extracted (reported as weight % of grinded hops pellets) as a function of both the extraction method and of the kind of extraction solvent used. Typical relative error in the quantity of extracted xanthohumol is estimated to be around 3%. The abbreviations are explained in the caption to Figure 3.

In order to present the results of the extraction efficiency of bitter acids and xanthohumol as a function of the type of extraction as well as of the kind of the solvent used in the extraction in a condensed manner, these results are presented not only numerically in Table S1 in Supplementary Materials but also graphically in Figure 3, Figure 4 and Figure 5. Due to the limited size of the space available, abbreviations and numbers are used in these figures to denote the type of the extraction method as well as the solvent used. These designations are described in Table 2 below. Mixed solvents (methanol–water, ethanol–water, and acetone–water) contained 50% (*v*/*v*) of water.

## 3. Results

The chromatographic analysis of the content of α-acids focused on the separate determination of the content of cohumulone as an important component of α-acids (in the Styrian Aurora variety of hops, cohumulone usually accounts for 22–26% of all α-acids) while the remaining α-acids (adhumulone and n-humulone) were determined together. At the same time, among the β-acids, the content of colupulone (which usually accounts for 50–55% of all β-acids), was determined separately from the other β-acids (adlupulone and n-lupulone). In the same chromatographic run, the content of xanthohumol as the most important representative of prenylated chalconoids in hops was also determined. To simplify the presentation of the results, only the total α-acid content, the total β-acid content, and the xanthohumol content are given here.

### 3.1. Extraction of A-Acids

On Figure 3, we graphically present the quantity of α-acids extracted (as the weight % of hop material from which α-acids were extracted, i.e., the mass of α-acids as was determined from HPLC chromatograms of extractants versus the mass of hop samples from which α-acids had been extracted, expressed in %) as a function of both the extraction method and the kind of the extraction solvent used.

#### 3.1.1. Extractions with Organic Solvents

As it can be seen from Figure 3, the highest content of α-acids was extracted by using the Soxhlet extraction method in combination with diethyl ether. Considering that in the case of Soxhlet extraction the solvent was recycled and that the ratio between the grinded hop pellets and the solvent used in the extraction differed from the ratio used in other kind of extractions, it is difficult to directly compare results of the Soxhlet extraction with other types of extraction. Instead, the results obtained with the Soxhlet extraction method using diethyl ether may serve more in a sense of a conditional benchmark for a successful extraction. Namely, diethyl ether is often used as a standard solvent for the extraction of bitter (α- and β-) acids as well as of xanthohumol from hops for analytical purposes.

Taking into account that bitter acids and xanthohumol are located on the surface of the hop tissue in lupulin glands, a longer contact time between the hops and the solvent may not be decisive for differences observed in the extraction efficiencies found for Soxhlet extraction and for other extraction methods. Namely, in such a case, diffusion processes occurring inside the tissues are not crucial for extraction mechanisms.

Considering use of ordinary solvents for the Sohxlet type extraction (only some of the most common solvents with relatively low boiling points were used in this study—hexane, ethyl acetate, methanol, and acetone), we were trying to elucidate a simple property of the solvent that would correlate well with the observed extraction efficiency [56]. One of the most frequently referred properties is polarity; however, in this case the correlation was not good. Better correlation was observed with Kamlet and Taft’s solvatochromic parameters b, and especially π*, but in both these cases diethyl ether was again the clear outlier in such a correlation.

When extraction was performed with the same solvents as used in the Soxhlet extraction but using orbital shaker extraction, the ordering (diethyl ether > hexane > ethyl acetate > methanol > acetone) was preserved although the extraction was a bit less successful (using parameters as described in Section 2.3 and Section 2.4). Considering that in orbital shaker extractions solvents with higher boiling points are usually used, we also included in the study for this type of extraction some other standard organic solvents and some of their mixtures with water in the case when solvents were fully miscible with water. Among these additionally included organic solvents, toluene was almost as efficient in the extraction of α-acids as hexane while ethanol was according to efficiency placed between ethyl acetate and methanol. Considering the use of mixed solvents containing water (methanol–water, ethanol–water, and acetone–water—all these solvents were prepared with 50 vol.% of water) we may see that the addition of water worsened extraction efficiency and that this efficiency was only minorly lost in the case of ethanol. On the other hand, efficiency was almost halved when methanol was replaced with a methanol–water mixture. Due to the unavailability of parameters used in the description of the physicochemical properties of the aforementioned mixtures of methanol, ethanol, and acetone with water, it was not possible to investigate possible correlations of these parameters for all the solvents used in orbital shaker extraction. Nevertheless, when mixed solvents were omitted from this analysis, it turned out—although correlations were still not perfect—that diethyl ether was again by far the most striking outlier in these correlations.

The extraction efficiency applying the same solvents as in the case of orbital shaker extraction was also tested with ultrasound-assisted extraction using an ultrasonic cleaning bath. Here, diethyl ether was again the most successful extraction solvent, followed by hexane, and then with a noticeable gap by toluene. The efficiency of extraction with ethyl acetate, methanol, acetone, and ethanol was practically the same and was only marginally lower than that with toluene. Concerning mixed solvents with water, the addition of water to methanol, acetone, and ethanol again diminished extraction efficiency, leaving the same order as in the case of orbital shaker extraction (ethanol > acetone > methanol). It is noteworthy to mention that in this case, a change in the extraction method from orbital shaker to the ultrasound-assisted extraction did not affect efficiency for aqueous acetone, while in the case of aqueous ethanol efficiency was only minorly diminished and it was significantly diminished for aqueous methanol. Analysis of the correlation of efficiency with physicochemical properties of solvents (again leaving out mixed solvents) showed over again that diethyl ether was the most noticeable outlier.

#### 3.1.2. Extractions with Deep Eutectic Solvents

The interesting question that appears is how successful can DESs be in the extraction of biologically important compounds from hops in comparison with organic solvents. Of course, due to the low vapor pressure of DESs, Soxhlet extraction with DESs was omitted from this comparison. On the other hand, the low vapor pressure of DESs enabled us to use DESs not only in orbital shaker and ultrasound-assisted extraction using an ultrasonic cleaning bath but also in a more aggressive ultrasonic homogenizer-assisted extraction. The results obtained in these three kinds of extraction are plotted on the right side of Figure 3. According to the literature data, in which comparisons of extraction efficiency for the extraction of (total) flavonoids, phenolic acids, and total phenolics from various natural sources using DESs on the one hand and classical solvents on the other hand were made [57], it can be expected that the DESs we prepared using alcohols, sugars, and organic acids as HBD components should probably be less efficient than methanol and ethanol in the extraction of α-acids (but also of β-acids and xanthohumol). These expectations were indeed met here. Yet, as one can easily notice from the figure, the composition of DESs is in this case extremely important. When choline chloride-based aqueous DESs were prepared with rather polar or even ionizable hydrogen donor components such as fructose, glucose, lactic acid, tartaric acid, glycerol, ethylene glycol, and urea and used in an orbital shaker or ultrasound (in an ultrasonic cleaning bath)-assisted extraction, the yield of extraction was very low—even lower than the lowest yield observed in the aforementioned methanol–water ultrasound (in ultrasonic cleaning bath)-assisted extraction. However, already the replacement of urea with less polar 1,3-dimethyl urea in DES as a hydrogen donor component considerably increased the yield of extraction (from 0.16 to 3.24 for the orbital shaker and from 0.21 to 3.42 for the ultrasound-assisted extraction) to become comparable with the one where acetone–water was used. Considering the well-known principle “like dissolves like”, it is then not surprising that DES prepared from choline chloride and phenol was the most successful DES tested here for the extraction of α-acids from hops. Moreover, the extraction efficiency of this DES can be, in some cases, under the same conditions even higher than that of diethyl ether.

The important feature that can be observed from Figure 3 is that it seems that more aggressive use of the ultrasound in extraction considerably increased extraction efficiency (the use of an ultrasonic homogenizer instead of an ultrasonic cleaning bath) of ChCl-TA DES (and also of some others, including ChCl-Glu) but eventually diminishes yield when ChCl-Phe is used. In that regard, the question arises as to which physicochemical properties of DESs are the ones that determine the direction of the effect of a more aggressive use of ultrasounds.

### 3.2. Extraction of B-Acids

In Figure 4, the amount of β-acids extracted (as the weight % of hop material from which β-acids were extracted) is shown as a function of the extraction method and the kind of extraction solvent used.

#### 3.2.1. Extractions with Organic Solvents

The extraction of β-acids by Soxhlet extraction shows a very similar pattern to that of α-acids when solvent efficiency is considered. The only minor difference is that the efficiency of ethyl acetate, methanol, and acetone is almost the same in the extraction of β-acids, while it slowly decreases from ethyl acetate to acetone in α-acids.

More differences when compared to the extraction of α-acids can be observed in the case of extraction using the orbital shaker. Here, five groups of efficiency can be observed. Again, the efficiency of extraction was the highest with diethyl ether, being followed with a distinctive difference by hexane, and then—again with a distinctive difference—by a rather unified group containing toluene, ethyl acetate, methanol, and ethanol. At this point, it is noteworthy to add that the use of orbital shaker extraction instead of Soxhlet extraction just minorly diminished the extraction efficiency of ethyl acetate and methanol in the extraction of β-acids. The fourth group comprised acetone, while the fifth group contained all three aqueous mixtures of organic solvents (methanol, ethanol, acetone). Although there is a notable difference in the extraction efficiency of these three mixtures (diminishing in the order ethanol > acetone > methanol), none of these three mixtures can be considered as an efficient solvent for the extraction of β-acids from hop cones. An important difference in extraction efficiency of these three aqueous mixtures when compared to their efficiency for the extraction of α-acids is that they are far less efficient in the extraction of β-acids than in the extraction of α-acids.

Further differences compared to the extraction of α-acids can be observed in the extraction with the orbital shaker. Here, five groups of efficiency values can be observed. Again, the extraction efficiency was highest with diethyl ether, followed with a significant difference by hexane, and then—again with a significant difference—by a fairly uniform group with toluene, ethyl acetate, methanol, and ethanol. It should be noted here that the use of orbital shaker extraction instead of Soxhlet extraction only slightly reduces the extraction efficiency of ethyl acetate and methanol in the extraction of β-acids. The fourth group contained acetone, while the fifth group contained all three aqueous mixtures of organic solvents (methanol, ethanol, and acetone). Although there is a remarkable difference in the extraction efficiency of these three mixtures (decreasing in the order ethanol > acetone > methanol), none of these three mixtures can be considered an efficient solvent for the extraction of β-acids from hop cones. An important difference in the extraction efficiency of these three aqueous mixtures compared to their efficiency in the extraction of α-acids is that they are much less efficient in the extraction of β-acids than in the extraction of α-acids.

For the ultrasound-assisted extraction with an ultrasonic cleaning bath, extraction with diethyl ether is again the most efficient, followed by that with hexane (which is about as efficient as Soxhlet extraction with hexane), followed by the group consisting of toluene, ethyl acetate, methanol, acetone, and ethanol. The replacement of OSE by UAE further increased the extraction efficiency of diethyl ether and hexane in the extraction of β-acids, as was already the case for α-acids. Among the less efficient solvents, the use of ultrasound-assisted extraction instead of orbital shaker extraction appeared to increase the extraction efficiency of toluene, ethyl acetate, methanol, and ethanol only slightly, but significantly for acetone. The lowest extraction efficiency was again observed for mixtures of methanol, ethanol, and acetone with water (again in the order: ethanol > acetone > methanol). In this case, the β-acids extraction efficiency using UAE instead of OSE was slightly higher for aqueous mixtures of ethanol and acetone, but lower for aqueous methanol.

#### 3.2.2. Extractions with Deep Eutectic Solvents

As with the extraction of α-acids, the use of DESs for the extraction of β-acids proved most promising for ChCl-Phe. Regardless of the extraction mode, this aqueous DES solution was by far the most efficient solvent among all the DESs tested. Its efficiency is comparable to that of diethyl ether and in the case of OSE even better than that of diethyl ether. It is worth noting that similar to the extraction of α-acids, some loss of the efficiency of ChCl-Phe was observed when the type of extraction technique was changed from OSE to UAE and then to UHE. Interestingly, the extraction efficiencies of all other DESs (except ChCl-DMU; this was similar to the use of aqueous acetone) were close to zero when OSE was used, but the efficiencies of these DESs were generally somewhat increased when UAE was used and further increased when UAE was applied. In all these cases, ChCl-BSA proved to be ineffective for extraction. Of the DES solvents with the exception of ChCl-Phe, only ChCl-TA showed extraction efficiencies roughly comparable to those of pure organic solvents—and even for ChCl-TA only when UHE was used.

### 3.3. Extraction of Xanthohumol

Figure 5 shows graphically the amount of xanthohumol extracted (in weight % of the hop material from which xanthohumol was extracted) as a function of the extraction method and the kind of extraction solvent used.

#### 3.3.1. Extractions with Organic Solvents

Looking at the extraction of biologically important compounds from hop cones, it is noticeable that while hexane is relatively successful in extracting bitter acids, it is ineffective in extracting xanthohumol. This observation holds true for all three types of extraction techniques tested for organic solvents. As for the use of diethyl ether for the extraction of xanthohumol, it can be said again that it is the most efficient solvent for this purpose. While the efficiency of diethyl ether is more than twice that of ethyl acetate, the second most efficient solvent in Soxhlet extraction, the change in extraction technique from Soxhlet to UAE almost halved the efficiency of diethyl ether. This change in extraction technique also affects the extraction efficiency of the other organic solvents used in Soxhlet extraction (ethyl acetate, methanol, acetone), although to a lesser extent. A comparison of the application of OSE and UAE shows comparable results, indicating the most efficient extraction with diethyl ether, followed by a gap (a group consisting of ethyl acetate, methanol, acetone, and ethanol), followed by about equally efficient mixtures of acetone and ethanol with water. Unlike the extraction of bitter acids, in the extraction of xanthohumol toluene is less efficient than mixtures of acetone and ethanol with water. The extraction efficiency of toluene is similar to that of aqueous methanol for OS, while UAE lowers the extraction efficiency of aqueous methanol, making toluene the more efficient solvent for the extraction of xanthohumol than aqueous methanol for UAE.

#### 3.3.2. Extractions with Deep Eutectic Solvents

Looking at the extraction efficiency of DESs for the extraction of xanthohumol, we find that the most efficient DES solvent is again ChCl-Phe and that its extraction efficiency for OSE and UAE is fully comparable with if not even higher than that of diethyl ether. Similar to the extraction of α- and β-acids, the extraction efficiency of ChCl-Phe for xanthohumol also depends to some extent on the technique used and decreases in the order OSE > UAE > UHE. An important difference in the use of DES for the extraction of xanthohumol and for the extraction of bitter acids is that ChCl-Phe is not the only DES tested agent that can be used quite successfully for the extraction of xanthohumol. The second most effective DES in this case is ChCl-DMU with an extraction efficiency of 82% of ChCl-Phe in the OSE technique, 62% in UAE, and 67% in UHE. This relative success of ChCl-DMU in extraction is much higher than its efficiency in the extraction of α- and β-acids. Among the other DESs tested, there are two other solvents whose extraction efficiency is close to that of organic solvents such as ethyl acetate, methanol, acetone, and ethanol. While the efficiency of ChCl-LA is almost the same as that of the aforementioned solvents, ChCl-EG achieves about 2/3 of their efficiency, making it comparable to mixtures of ethanol and acetone with water. In general, the other DESs are also more efficient in the extraction of xanthohumol than in the extraction of bitter acids. For example, the efficiency of ChCl-TA and ChCl-Gly is on average (depending on the extraction technique) comparable to that of aqueous methanol. However, it is somewhat surprising that ChCl-BSA, which contains an aromatic ring, is among the DESs with the lowest extraction efficiency. It is possible that in this case, the presence of a strongly ionized sulfonic group prevents ChCl-BSA from being an efficient solvent for xanthohumol, although a small degree of ionization should not be an obstacle per se, as has been demonstrated for ChCl-LA and ChCl-TA.

### 3.4. Physicochemical Properties of DES Solvents

#### 3.4.1. Density

**Figure 6:**
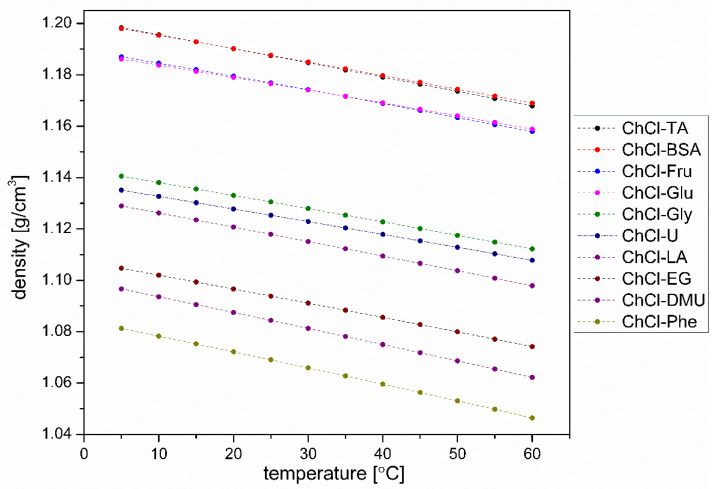
Densities of DES solvents used in extraction as a function of temperature.

The measured densities show (Figure 6) that—as expected—the densities of all prepared concentrated aqueous solutions decrease with the increase of temperature. As it can be seen, this decrease is fairly regular and, to a first approximation, linear with very similar slopes. The highest densities are those of ChCl-TA and ChCl-BSA, followed by ChCl-Fru and ChCl-Glu. For the latter, one would expect identical or nearly identical densities at given temperatures, and this expectation is met here. It is difficult to say whether the minimal differences between them are due to the small differences in the structure of the two monosaccharides or whether it is an experimental error (small differences in the composition of the prepared solvent). Although both solvents were prepared with the usual care, we did not check the content of water in these monosaccharides, so we cannot exclude the possibility that the observed minimal differences are at least partly due to a slightly different composition of the two solvents.

ChCl-Gly, ChCl-U, and ChCl-LA follow with a notable gap, which could also be a partial result of the stronger hydration in the four densest DESs. Here, the hydrogen bond donating components contain methyl groups, which cannot contribute to hydrophilic hydration and thus to the very effective packing of water molecules in space, and, moreover, the relative content of heavier atoms (oxygen) is lower than in the densest DESs (note that ChCl-BSA—although it contains a benzene ring—also contains a sulfur atom and that the sulfonic group is almost completely ionized). Moreover, the lactic acid used for the preparation of ChCl-LA was declared to have a purity of 88–92% (*w*/*w*), which further complicates the comparison of the properties of this DES with those of other DESs.

The group of DESs that have the lowest densities are ChCl-EG, ChCl-DMU, and ChCl-Phe (listed in decreasing order of density). Although there could be other correlations, this order—among several other possible correlations—is also consistent with the increasing hydrophobicity of the HBD constituents, phenol being the most hydrophobic among them. Namely, while pure ethylene glycol is completely miscible with water at room temperature, one can dissolve about 800 g of dimethylurea in 1 L of water, but only about 80 g of phenol.

It is beyond the scope of this study to go into a more detailed analysis of the densities; we simply aim to draw the readers’ attention to the interplay of the hydrophilic/hydrophobic character of the DESs studied and their possible effects on the density of these solvents.

#### 3.4.2. Speed of Sound

**Figure 7:**
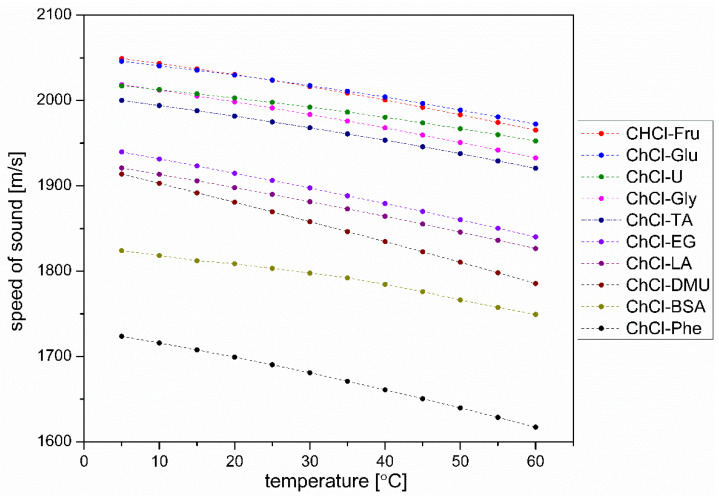
Speed of sound propagation in DES solvents used in extraction as a function of temperature.

**Figure 8:**
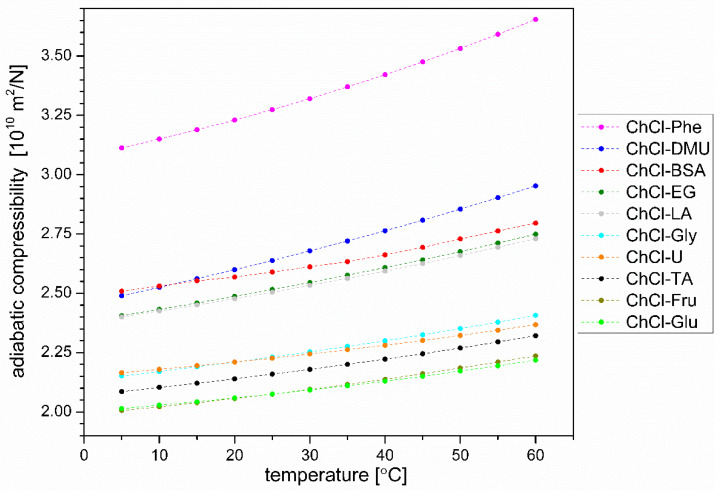
Adiabatic compressibility of DES solvents used in extraction as a function of temperature.

The speed of sound propagation in liquids is equal to the square root of the ratio between the bulk modulus (which is the reciprocal of adiabatic compressibility) and the density of the liquid. Considering that the bulk modulus also depends on the distances between the particles in the liquids and that these distances are related to the density, it is not trivial to predict the speed of sound in liquids with quite similar compositions. Therefore, only the experimentally measured temperature dependence of the sound velocity in the prepared DESs is shown in Figure 7. These values are then used for the adiabatic compressibility calculations shown in Figure 8.

#### 3.4.3. Adiabatic Compressibility

Isothermal compressibility of liquids is an important piece of information whenever fluid flow is considered. The numerical values of the isothermal compressibilities of liquids are almost equal to the numerical values of adabatic compressibilities; however, using modern instrumentation the later ones are far more easily determined just from the measured density of the liquid and the speed of sound propagation in the liquid.

As shown in Figure 8, adiabatic compressibility is the lowest for DES-based solvents where relatively strong hydration of HBD is expected (ChCl-Glu, ChCl-Fru, ChCl-TA), increasing then in an approximate order (from lowest to highest) ChCl-U ≈ ChCl-Gly < ChCl-LA ≈ ChCl-EG < ChCl-BSA < ChCl-DMU << ChCl-Phe. It is the adiabatic compressibility of ChCl-Phe that is most different from the others. Because of the relatively low water content in DES-based solvents (at least 25% (*w*/*w*), slightly more in solvents where water was already present in the components forming DES), it is difficult to assert beyond doubt that the main contributor to the differences in adiabatic compressibility is extensive hydrophobic hydration. Nevertheless, the observed sequence is consistent with the theoretical finding [58] that compressibility in liquids increases with the increase of the surface area where hydrophobic hydration takes place. Following this finding, one could interpret that the relatively high adiabatic compressibility of ChCl-Phe is due to the large surface area where the hydrophobic surface (in this case the aromatic ring) is in direct contact with water. Note that although the weight fraction of water in ChCl-Phe is only 25%, there are still 7.8 molecules of water available for the hydration of one molecule of choline chloride and three molecules of phenol (i.e., on average, almost two molecules of water to one rather nonpolar molecule). Extending our interpretation further, we can attribute the observed high extraction efficiency of ChCl-Phe to the presence of extensive hydrophobic surfaces not present in other solvents. The second highest compressibility was observed for ChCl-DMU; it was also the second best DES-based solvent tested in our study. In contrast, most of the solvents with the lowest extraction efficiency are found among the solvents with low compressibility (ChCl-Fru, ChCl-Glu, ChCl-U). In the group of solvents with the lowest extraction efficiency, ChCl-BSA is an exception in terms of compressibility. Namely, the aromatic ring of benzenesulfonic acid is hydrophobic, but a highly polar (the degree of ionization is almost 100%) sulfonic group is attached to this hydrophobic part of the molecule. The water molecules around the sulfonic group are strongly bound to the charged sulfonic group (hydrophilic hydration), which significantly limits the possibility of forming a large surface around the aromatic ring where hydrophobic hydration could take place [59]. While this surface area around the aromatic ring might be large enough to accommodate some water molecules arranging around the ring in a hydrophobic manner and thus increase the compressibility of the solvent, such geometry (a highly polar group in the vicinity of a small hydrophobic surface) does not allow attractive interactions of this aromatic ring with hop polyphenols (presumable attraction through π–π interactions). Due to the lack of such interactions, ChCl-BSA may not be an efficient solvent for the extractions studied.

The other four solvents not mentioned above (ChCl-LA, ChCl-TA, ChCl-EG, and ChCl-Gly) have been used successfully to some extent for the extraction of polyphenols from hops, and their compressibilities are also somewhere in the middle between the most and least efficient extraction solvents. A more detailed analysis of the hydration around these HBD components is omitted here due to a lack of suitable information.

#### 3.4.4. Viscosity

**Figure 9:**
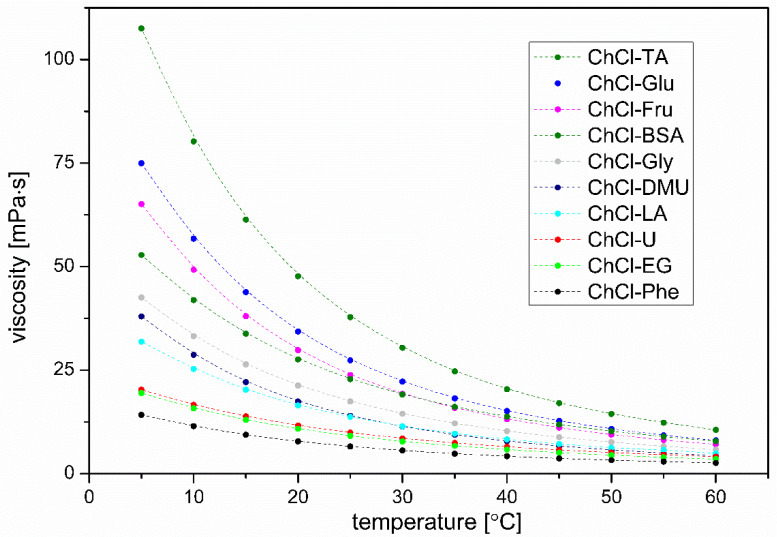
Viscosities of DES solvents used in extraction as a function of temperature.

In the case of electrolytes, it is known that the stronger the hydrophilic hydration around the ions, the more viscous the solution (keeping the molar concentration of ions in solutions of different electrolytes constant) [60]. Further, some correlation between the strength of hydrophilic hydration and viscosity can also be observed for other polar molecules, such as glycosaminoglycans [61]. Therefore, it is not surprising that ChCl-TA, ChCl-Glu, ChCl-Fru, and ChCl-BSA exhibit the highest viscosities among the solvents studied (Figure 9). Regarding the viscosity of ChCl-Glu and ChCl-Fru, which have almost the same structure (and their density and compressibility are also practically identical), we found that ChCl-Glu is slightly more viscous than ChCl-Fru. This finding may be somewhat unexpected, but it must be said that (at the same temperature and concentration) pure aqueous glucose solutions are also somewhat more viscous than fructose solutions [62]. We can further speculate that these small differences in hydration may be an additional reason (in addition to possible experimental errors) why the density and adiabatic compressibility of ChCl-Glu and CHCl-Fru are not completely identical.

In the case of ChCl-Phe, low viscosity is expected due to the aforementioned predominant hydrophobic hydration of the HBD component.

#### 3.4.5. Structural versus Dilution Role of Water during Preparation of DES

Our presumption was that the two preparation procedures we used would result in an aqueous DES-based solvent with equivalent physicochemical properties. This assumption was based on the consideration that the interactions among components forming concentrated aqueous DES solutions are relatively weak when compared with the thermal energy of the molecules in the system subjected to a temperature of 80 °C. Thus, if the system remains at the elevated temperature for a sufficiently long time, it should be possible to achieve the same thermodynamically stable state regardless of the procedure of preparation.

Two batches of concentrated aqueous ChCl-Phe were prepared by heating and stirring. In the first batch, pure (anhydrous) ChCl-Phe was first prepared and then water was added. In the second batch, water was added to the mixture of choline chloride and phenol at the very beginning. The temperature dependence of density, speed of sound propagation, adiabatic compressibility, and viscosity were the same; in this case, the same within the differences that normally occur when the solvent is prepared by the same procedure but in different batches. Moreover, no significant difference was found between the extraction efficiency of the two batches. Therefore, for our purposes, both preparation procedures are equivalent.

## 4. Discussion

The results of the individual experiments have already been discussed where the results were presented, while the broader perspective of this study will be addressed here.

### 4.1. Extraction Efficiency of DES Solvents

The basic question was whether it is possible to prepare DES solvents comparable in extraction efficiency to the extraction efficiency of classical organic solvents used for the extraction of polyphenols from hops, and how the choice of extraction method affects the extraction efficiency in this particular case.

As can be seen from Figure 3, Figure 4 and Figure 5, it is the choice of DES solvent that has the greatest impact on the efficiency of the extraction. Namely, with the advent of DES solvents it has become possible to prepare liquid solvents containing a high concentration of molecules capable of dissolving the desired solutes, although the solvent molecules that enable good solubility of the solutes are not present in liquid form in the pure state, nor is it possible (or very difficult) to prepare mixed solvents containing a high concentration of such solvent molecules. In our case, this was nicely demonstrated by the example of phenol. As shown by the measurements of the physicochemical properties of the prepared DES-based solvents, the hydrophobic nature (and most likely the chemical similarity) of phenol is the crucial factor enabling high extraction efficiency of DES-Phe in the extraction of polyphenols. In its pure form, phenol is a crystalline solid with a melting point of about 41 °C at atmospheric pressure, whereas it forms a single-phase system with water only at temperatures above 67 °C [63]. These temperatures are too high to be used for the extraction of poylphenols from hops. On the other hand, the formation of DES between choline chloride and phenol in a molar ratio of 1:3 with the addition of some water (in our case 25% *w*/*w*) allows the formation of a liquid solvent containing 50.2% *w*/*w* phenol at room temperature. Such a high phenol content in mixed solvents is difficult to achieve even with organic solvents.

While the extraction efficiency of ChCl-Phe is quite comparable to that of diethyl ether (see above), this is less true for the other DES solvents studied here. A certain exception is ChCl-DMU, whose extraction efficiency of xanthohumol is comparable to that of diethyl ether in OSE.

As for the comparison of our work with previous studies on the extraction of bioactive compounds from hops, it is difficult to compare them in detail because neither the hop samples nor the extraction procedures were identical. However, if we compare the results of our study with the one carried out by Lakka et al. [48], in which the extraction efficiency for the extraction of total polyphenols with L-alanine glycerol DESs was similar to that they observed with 60% methanol or 60% ethanol, with the one we observed (results for the extraction of xanthohumol with DESs, 50% methanol and 50% ethanol), then we can probably assume that ChCl-LA, ChCl-EG are at least as similarly effective as L-alanine glycerol, while ChCl-DMU and ChCl-Phe are superior for the extraction of xanthohumol from hops. In the works of Grudniewska et al. [49,50], similar extractants based on DES were used for the extraction of xanthohumol from spent hops, and we can say that ChCl-Phe and most probably also ChCl-DMU, considering only the extraction efficiency, are better extractants than those used by them. However, a comparison of their results with ours shows that their extractant ChCl-Gly, prepared in a molar ratio of 1:2, is better for the extraction of xanthohumol than ours, prepared in a molar ratio of 1:1. A comparison with the results of the study carried out by Macchioni et al. [51] shows that our ChCl-Phe extractant is more effective in the extraction of bitter acids than the extractants used in their work. Although the efficiency of the extractant LA-sucrose used by them is lower than that of our ChCl-Phe, LA-sucrose still seems to outperform all other DES-based extractants used by us. Interestingly, in our case, the efficiency of the ChCl-LA extractant was marginal in the extraction of α- and β-acids. On the other hand, only a rough comparison can be made with their results. Indeed, they used different hop varieties (the content of α- and β-acids in the different varieties can vary considerably) and applied a longer and combined extraction method (30 min stirring phase followed by a phase in which the samples were ultrasonicated for 30 min). They also repeated the extraction procedure with the centrifugate obtained after centrifugation, and only the final results (after two extraction cycles) are reported.

### 4.2. Choice of the Optimal Extraction Technique

In Figure 3, Figure 4 and Figure 5, we find several interesting features related to the choice of the optimal extraction technique.

If we start with the use of ChCl-Phe, we can observe that among the techniques used, it was most efficient in OSE extraction, followed by UAE, and least efficient in UHE. This observation is valid for the extraction of α- and β-acids as well as for xanthohumol.

The observation for the use of ChCl-DMU is a little different. While ChCl-DMU behaves similarly to ChCl-Phe in the extraction of xanthohumol (where its efficiency is not drastically lower than that of ChCl-Phe and also the efficiency decreases in the order OSE > UAE > UHE), the situation is different for α- and β-acids. Here, the efficiency of ChCl-DMU is about the same in the case of OSE and UAE, but the use of a more energetic ultrasound (UHE) increases the extraction efficiency somewhat.

The effect of increased efficiency when going from OSE and UAE to UHE is even more pronounced for ChCl-TA and is especially true for the extraction of α- and β-acids. The last observation may be related to the fact that also α- and β-acids are more hydrophobic in nature than xanthohumol. A trend similar to that observed for ChCl-TA can also be observed for other DES-based solvents with low compressibility (ChCl-Glu, ChCl-Fru, ChCl-Gly, ChCl-U). Although their extraction efficiencies are low for all extraction techniques and for all extracted compounds, one can still observe the trend that the extraction efficiency increases in the direction from OSE to UHE.

In view of the above, ultrasound-assisted extraction seems to be particularly useful when the compressibility of the solvents is low. On the other hand, it seems that replacing orbital shaker extraction with ultrasound-assisted extraction (while reducing extraction time) is not fruitful in cases where solvents with high compressibility are used. Probably, the mechanical vibration induced by the ultrasonic wave is lower for solvents with higher compressibility and therefore less effective for extraction. As far as we know, there are no published studies addressing the relationship between extraction efficiency and solvent compressibility in ultrasound-assisted extraction.

### 4.3. Possible Improvements of Extractions Using DES-Based Solvents

Although the successful preparation of a DES-based solvent that has a similar extraction efficiency to the best organic solvent for the given compound could be a success in itself in some cases, this was only a partial objective of our study, just to show that the diversity of components from which DES-solvents can be prepared allows a good adjustment of the extraction power of DES-based solvents. Indeed, we are aware that extraction with DES-based solvents is in the vast majority of cases still far from being useful to be applied in practice. In addition, even if the individual components from which DES-solvents are prepared are in many cases harmless, this is not necessarily true for the prepared DES [64]. Our broader aim, in line with the appeal in the conclusion of the above-mentioned review [3], was to contribute to a better understanding of the relationship between the physicochemical properties of DES-based solvents and their applications. Considering the rather polar nature of the most commonly used components of DESs and the hydrophobicity of phenols in hop cones, the role of water—which is usually part of DES-based solvents in the extraction of bioactive compounds—can be very important in such cases [58]. Therefore, an important part of this study was devoted to measuring the physicochemical properties of the prepared DES-based solvents (in this case, among others, to adiabatic compressibility, which is a good approximation of isothermal compressibility in liquids, the latter being related to the structure of liquids).

In this respect, this study is the starting point for more comprehensive research addressing the role of water and the role of ions in DES-based systems [65]. This topic has only recently been addressed on a theoretical basis [66], although the importance of hydrogen bonding in both these [3] as well as in other mixed hydrophilic–hydrophobic systems [67] is widely recognized. Given our experience with the influence of the nature of counterions on the properties of aqueous systems [68,69,70] and the influence of ions on the strength of hydrogen bonds [71], we have already initiated an experimental study addressing the role of the nature of counterions on the physicochemical properties and practical applications in DES choline-based systems.

A better understanding of the role of water and solute hydration in DES-based aqueous systems will hopefully contribute to a more rational design of separation and extraction procedures. One such possibility is the use of two-phase systems consisting of aqueous and hydrophobic DES phases [72], where the subtle differences between hydrophilicity and hydrophobicity of the two phases and solutes determine the separation efficiency. Such an attempt was not made in this study, but remains one of the future challenges.

## 5. Conclusions

In this study, it was shown that the type of extraction can affect the efficiency of the extraction of bitter acids and xanthomunol from hop cones. However, a more important factor determining extraction efficiency is the molecular structure of the solvent, and the old principle “like dissolves like” is still a good guide for initial solvent selection experiments. Nevertheless, all effects are difficult to predict, and to better understand such systems, one should probably resort to more complex theoretical approaches, such as the use of molecular dynamics simulations [73].

It is well known that several physicochemical properties of solvents should usually be considered in advance when choosing the optimal extraction technique (e.g., boiling point, vapor pressure, density, viscosity, polarity, p*K*_a_ value) [12,74]. According to the collected evidence, the compressibility of the solvent also seems to be one of the factors that should be considered when searching for the optimal extraction technique.

## Figures and Tables

**Table 1:** Composition and the molar ratio of the DESs used in this study.

Abbreviation	Composition of DES ^1^	Molar Ratio
ChCl-Glu	Choline chloride:Glucose:Water	2:1:1
ChCl-Fruc	Choline chloride:Fructose:Water	2:1:1
ChCl-LA	Choline chloride:Lactic acid	1:1
ChCl-TA	Choline chloride:Tartaric acid	2:1
ChCl-Gly	Choline chloride:Glycerol	1:1
ChCl-EG	Choline chloride:Ethylene glycol	1:2
ChCl-U	Choline chloride:Urea	1:1
ChCl-DMU	Choline chloride:1,3-dimethylurea	1:2
ChCl-Phe	Choline chloride:Phenol	1:3
ChCl-BSA	Choline chloride:Benzenesulfonic acid	1:1

^1^ To the denoted composition, an extra amount of ultrapure water (corresponding to 1/3 of DES mass) was added. The final solutions therefore contained 75% of DES and 25% of “extra” water (*w*/*w*).

**Table 2:** Designations of type of extractions and solvents used presented in Figure 3, Figure 4 and Figure 5.

	Group 1	Group 2	Group 3	Group 4	Group 5	Group 6
Type of solvents:	Organic	Organic	Organic	DES	DES	DES
Type of extraction:	Soxhlet	Orbital shaker	UAE *	Orbital shaker	UAE *	UHE *
Abbreviation used:	OSs-Soxhlet	OSs-OSE	OSs-UAE	DESs-OSE	DESs-UAE	DESs-UHE
Samples	1 diethyl ether 2 hexane 3 ethyl acetate 4 methanol 5 acetone	6 toluene 7 diethyl ether 8 hexane 9 ethyl acetate 10 methanol 11 acetone 12 ethanol 13 methanol–water 14 ethanol–water 15 acetone–water	16 toluene 17 diethyl ether 18 hexane 19 ethyl acetate 20 methanol 21 acetone 22 ethanol 23 methanol–water 24 ethanol–water 25 acetone–water	26 ChCl-Glu 27 ChCl-Fru 28 ChCl-LA 29 ChCl-TA 30 ChCl-Gly 31 ChCl-EG 32 ChCl-U 33 ChCl-DMU 34 ChCl-Phe 35 ChCl-BSA	36 ChCl-Glu 37 ChCl-Fru 38 ChCl-LA 39 ChCl-TA 40 ChCl-Gly 41 ChCl-EG 42 ChCl-U 43 ChCl-DMU 44 ChCl-Phe 45 ChCl-BSA	46 ChCl-Glu 47 ChCl-Fru 48 ChCl-LA 49 ChCl-TA 50 ChCl-Gly 51 ChCl-EG 52 ChCl-U 53 ChCl-DMU 54 ChCl-Phe 55 ChCl-BSA

* UAE—Ultrasound-assisted extraction using ultrasonic cleaning bath; UHE—Ultrasonic homogenizer extraction.

## Data Availability

Data are contained within the article and Supplementary Materials.

## Supplementary Materials

The following supporting information can be downloaded at: https://www.mdpi.com/article/10.3390/plants12162890/s1, Figure S1a–c: Density, viscosity, and adiabatic compressibility of concentrated ChCl-Gly aqueous DES mixture as a function of temperature and the kind of preparation. Figure S2a–c: Density, viscosity, and adiabatic compressibility of concentrated ChCl-EG aqueous DES mixture as a function of temperature and the kind of preparation. Figure S3a–c: Density, viscosity, and adiabatic compressibility of concentrated ChCl-DMU aqueous DES mixture as a function of temperature and the kind of preparation. Figure S4a–c: Density, viscosity, and adiabatic compressibility of concentrated ChCl-Phe aqueous DES mixture as a function of temperature and the kind of preparation. Table S1: Quantity of α-acids, β-acids, and xanthohumol extracted. Table S2: Statistical differences observed in extraction of α-acids, β-acids, and xanthohumol.
