# Supplementary material for: Extraction of Polyphenols from Slovenian Hop (Humulus lupulus L.) Aurora Variety Using Deep Eutectic Solvents: Choice of the Extraction Method vs. Structure of the Solvent
Source: Plants (Basel). 2023 Aug 8;12(16):2890. doi: 10.3390/plants12162890 (PMC10459876; doi:10.3390/plants12162890)
Supplement: Supplementary file 1 [file plants-12-02890-s001.zip › plants-2461173-supplementary.pdf]

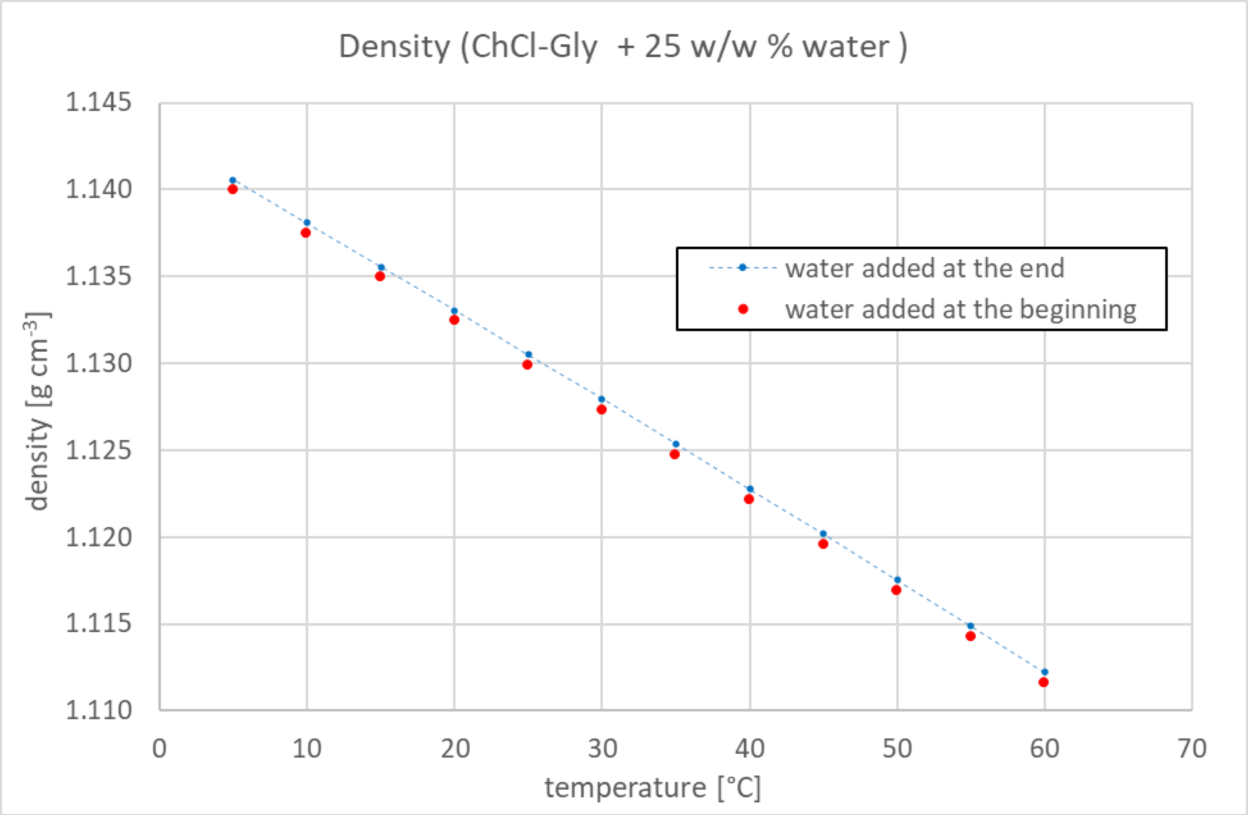

Figure S1a. Density of concentrated ChCl-Gly (Table 1) aqueous DES mixture as a function of temperature and the kind of preparation (time of adding water to DES). The samples were prepared by different persons.

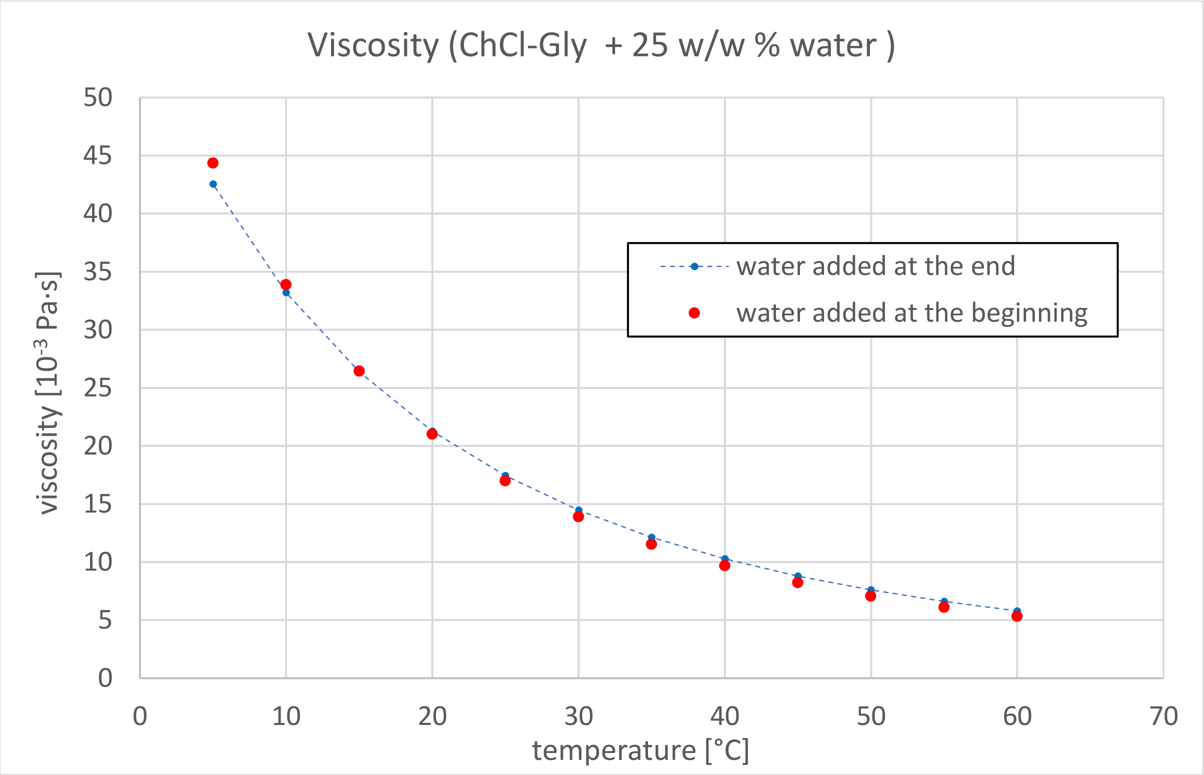

Figure S1b. Viscosity of concentrated ChCl-Gly (Table 1) aqueous DES mixture as a function of temperature and the kind of preparation (time of adding water to DES). The samples were prepared by different persons.

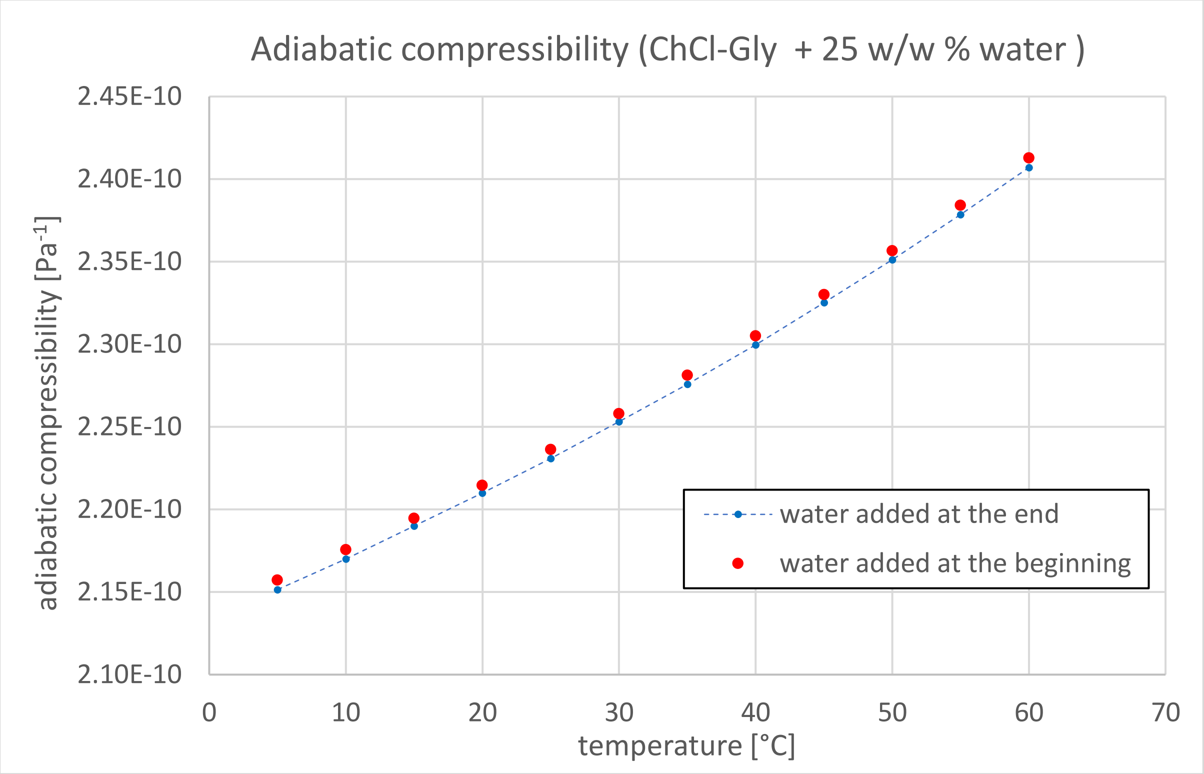

Figure S1c. Adiabatic compressibility of concentrated ChCl-Gly (Table 1) aqueous DES mixture as a function of temperature and the kind of preparation (time of adding water to DES). The samples were prepared by different persons.

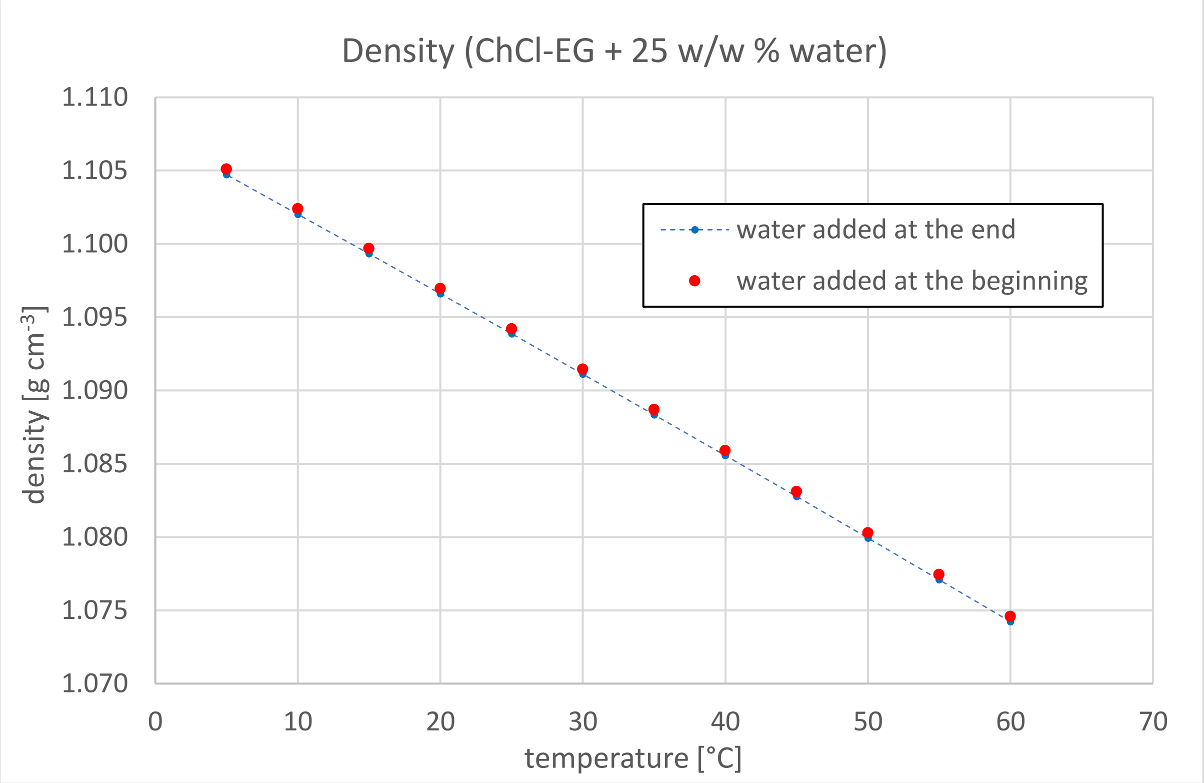

Figure S2a. Density of concentrated ChCl-EG (Table 1) aqueous DES mixture as a function of temperature and the kind of preparation (time of adding water to DES). The samples were prepared by different persons.

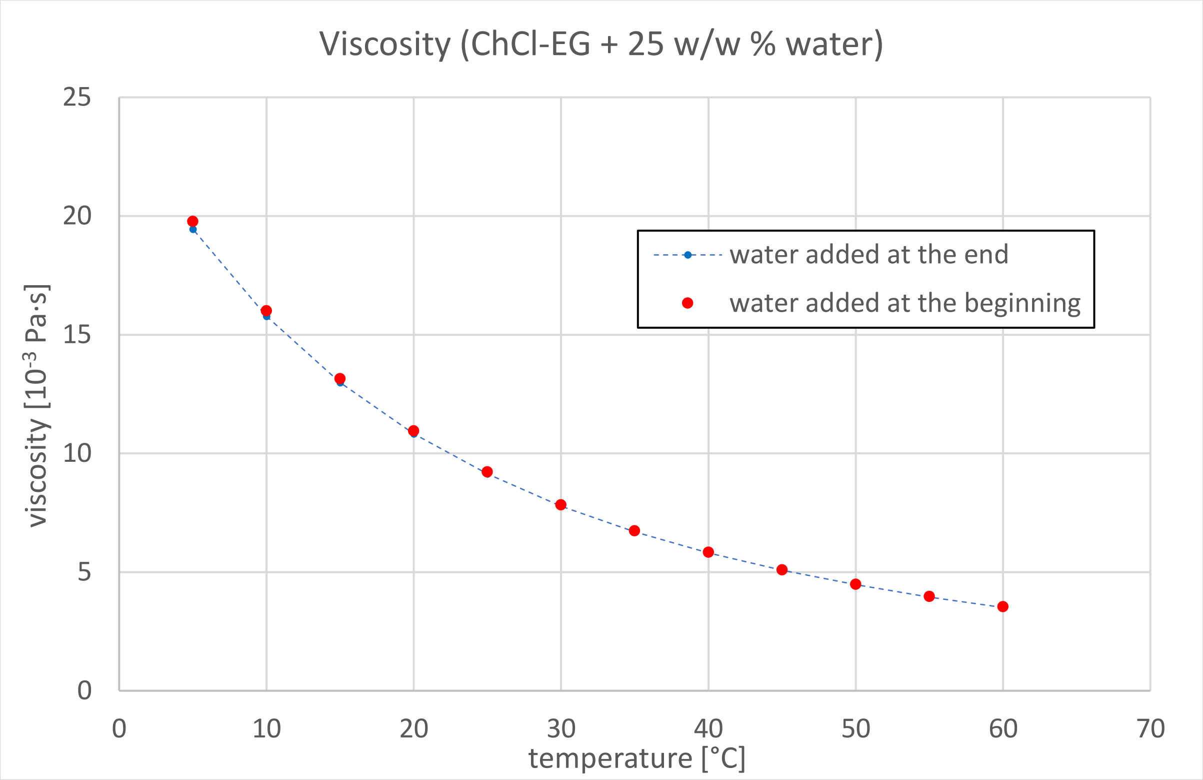

Figure S2b. Viscosity of concentrated ChCl-EG (Table 1) aqueous DES mixture as a function of temperature and the kind of preparation (time of adding water to DES). The samples were prepared by different persons.

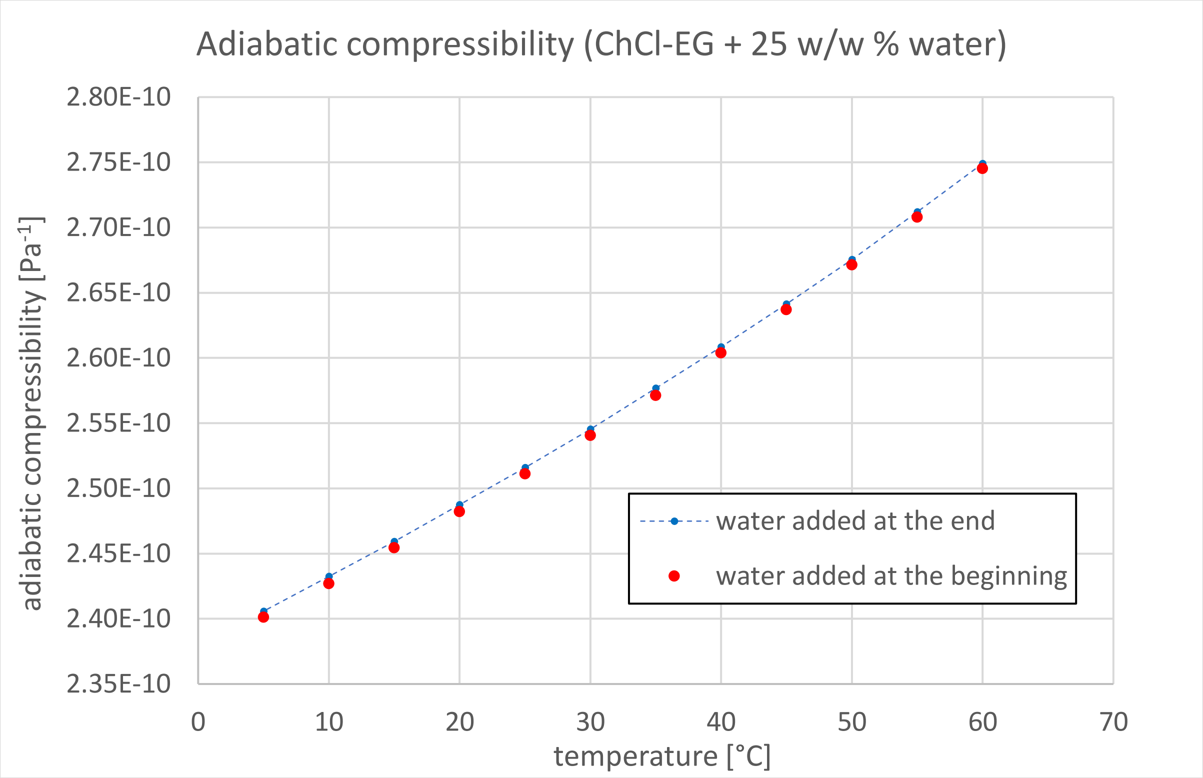

Figure S2c. Adiabatic compressibility of concentrated ChCl-EG (Table 1) aqueous DES mixture as a function of temperature and the kind of preparation (time of adding water to DES). The samples were prepared by different persons.

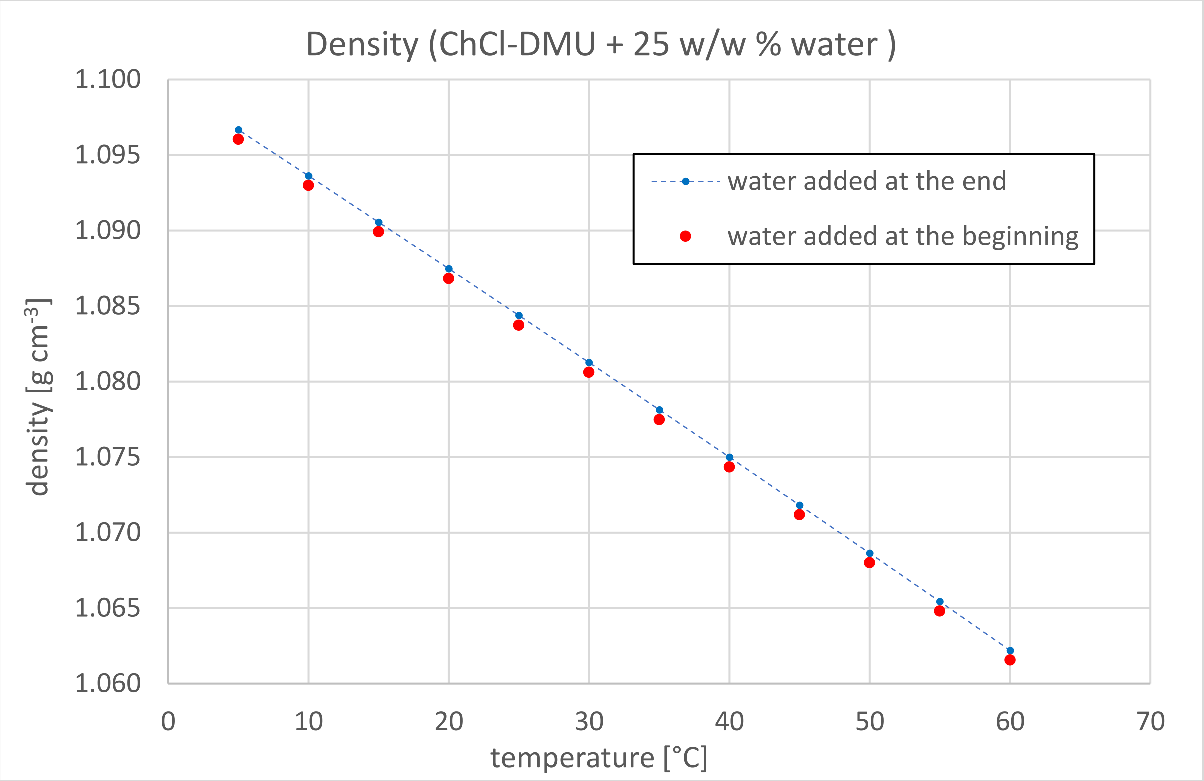

Figure S3a. Density of concentrated ChCl-DMU (Table 1) aqueous DES mixture as a function of temperature and the kind of preparation (time of adding water to DES). The samples were prepared by different persons.

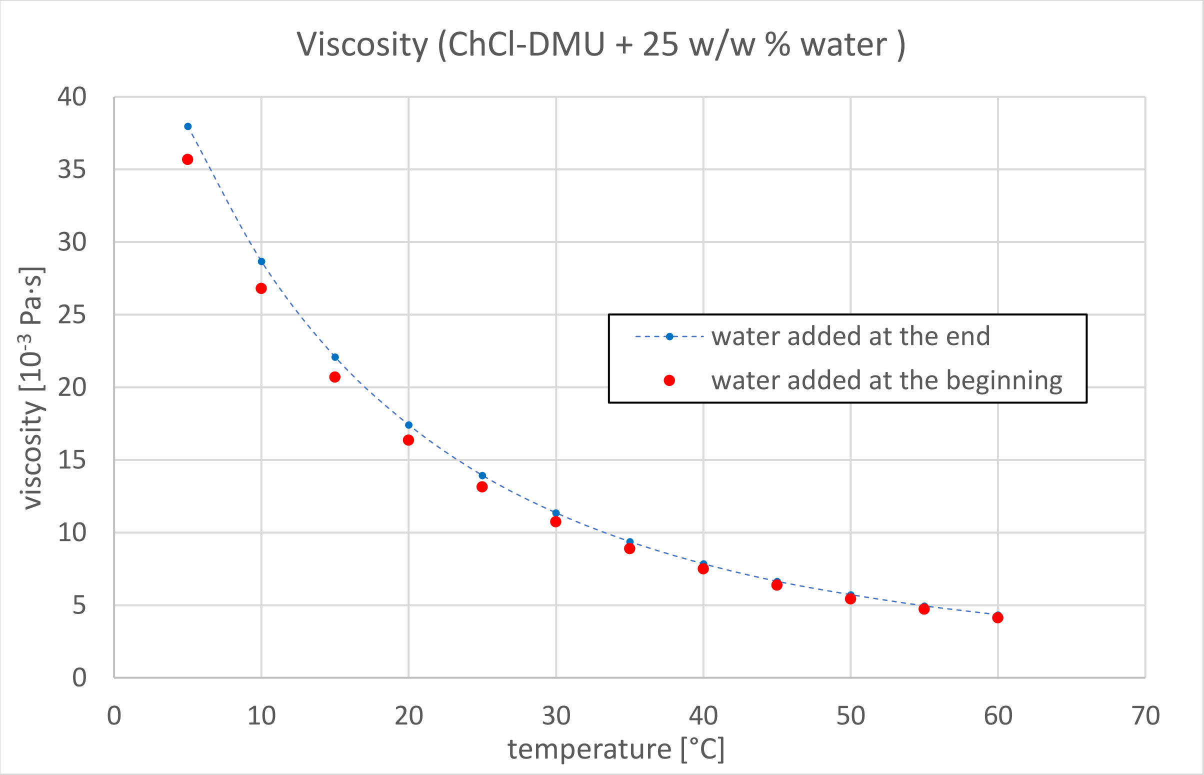

Figure S3b. Viscosity of concentrated ChCl-DMU (Table 1) aqueous DES mixture as a function of temperature and the kind of preparation (time of adding water to DES). The samples were prepared by different persons.

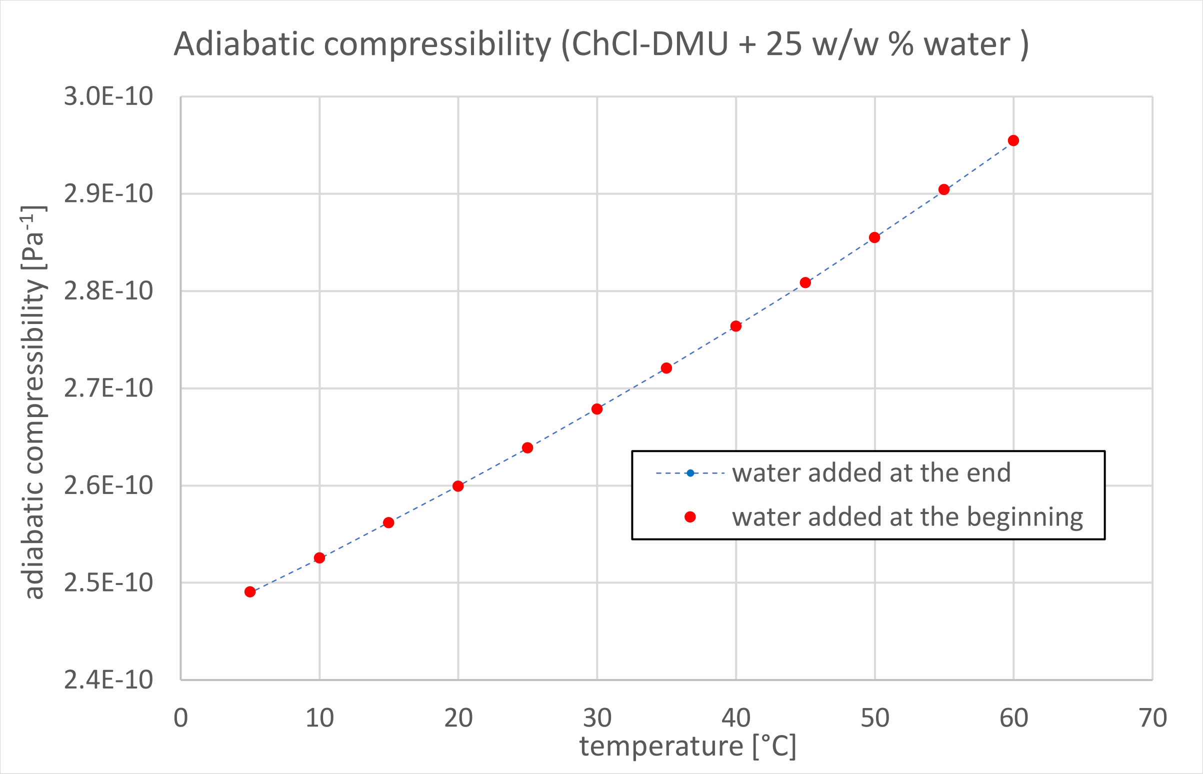

Figure S3c. Adiabatic compressibility of concentrated ChCl-DMU (Table 1) aqueous DES mixture as a function of temperature and the kind of preparation (time of adding water to DES). The samples were prepared by different persons.

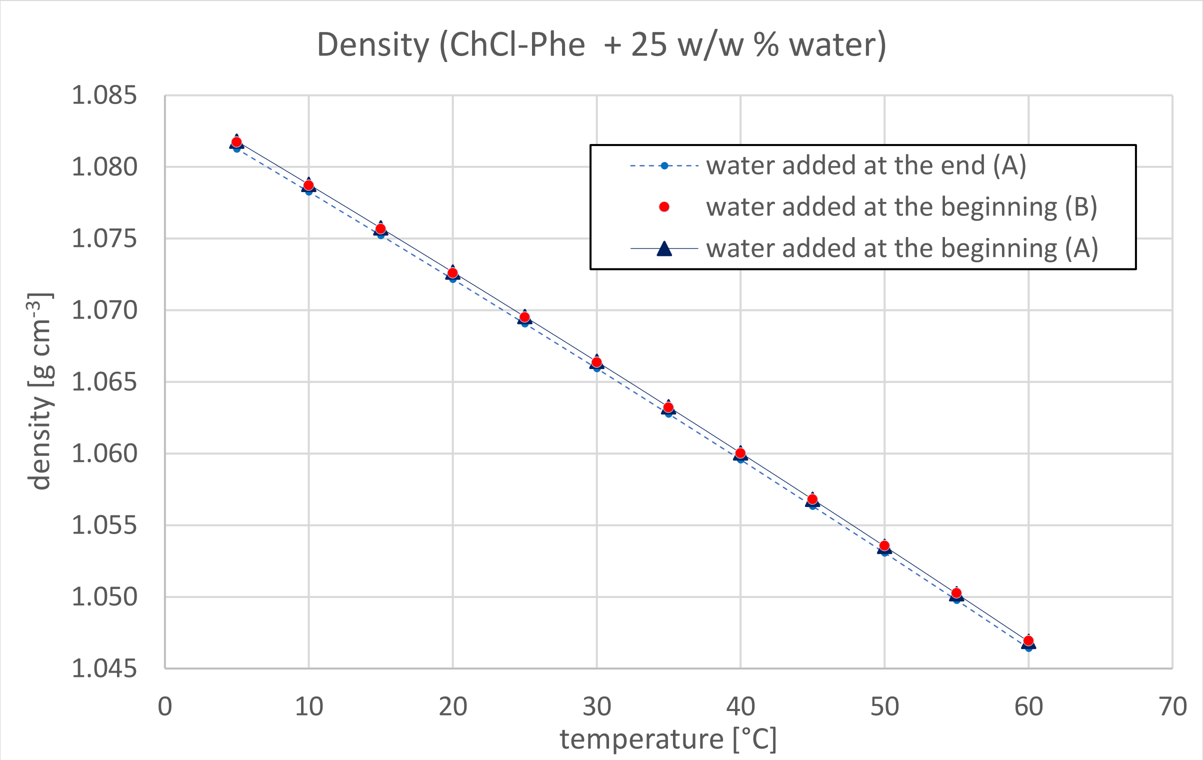

Figure S4a. Density of concentrated ChCl-Phe (Table 1) aqueous DES mixture as a function of temperature and the kind of preparation (time of adding water to DES). The samples were prepared by different persons (A and B).

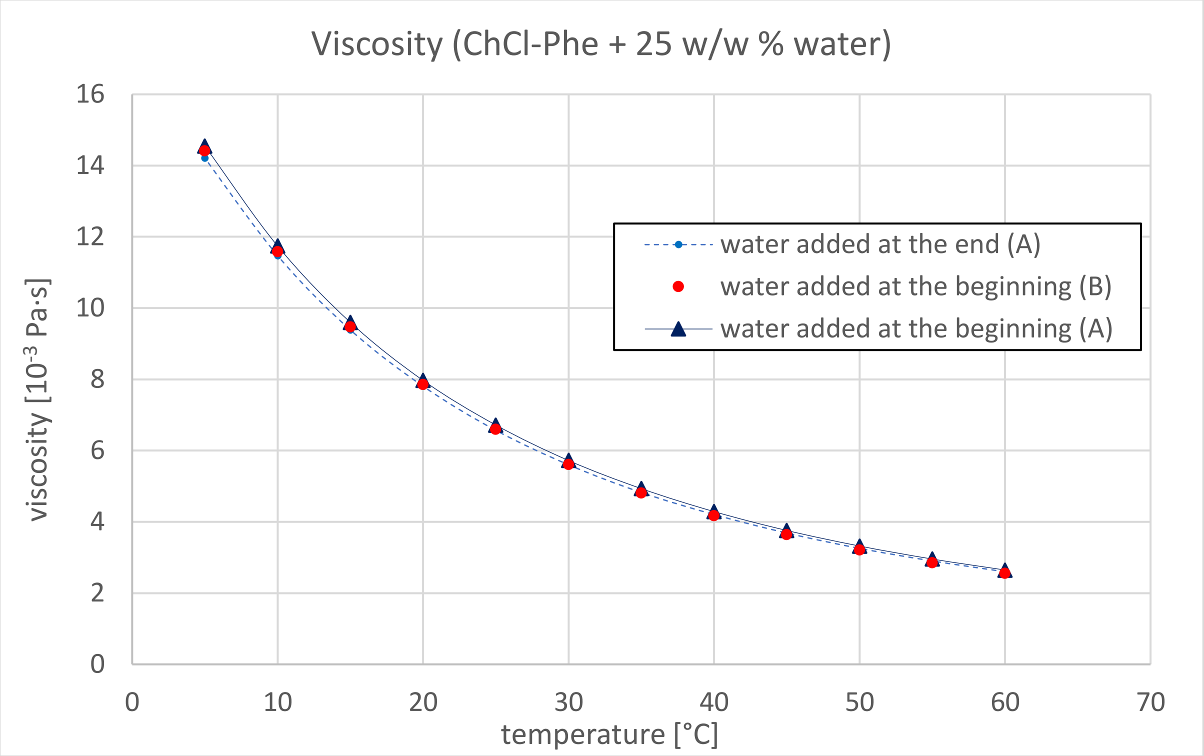

Figure S4b. Viscosity of concentrated ChCl-Phe (Table 1) aqueous DES mixture as a function of temperature and the kind of preparation (time of adding water to DES). The samples were prepared by different persons (A and B).

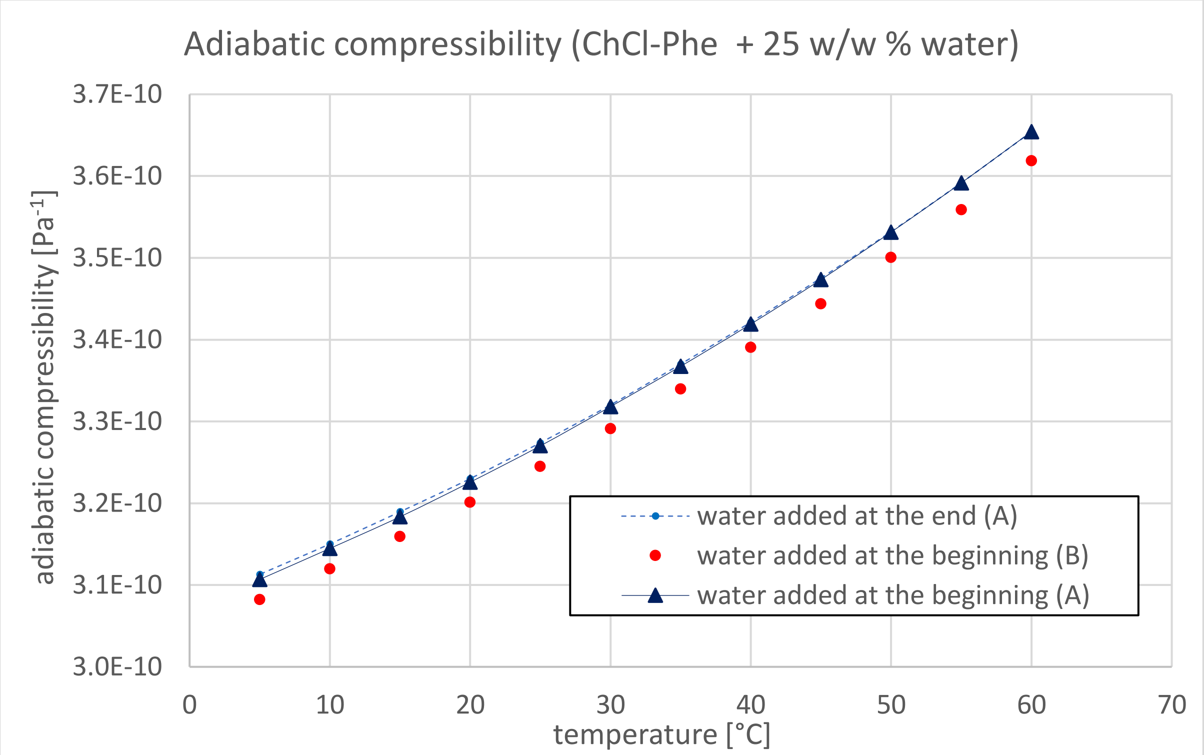

Figure S4c. Adiabatic compressibility of concentrated ChCl-Phe (Table 1) aqueous DES mixture as a function of temperature and the kind of preparation (time of adding water to DES). The samples were prepared by different persons (A and B).

Table S1. Quantity of α-acids, β-acids, and xanthohumol extracted (reported as weight % of grinded hops pellets) as a function of both of extraction method and of kind of the extraction solvent used.

sample Nr	solvent	extraction method	α-acids (total)	β-acids (total)	xanthohumol
1	diethyl ether	Soxhlet apparatus	15.661	7.640	1.389
2	hexane	Soxhlet apparatus	10.778	5.267	0.008
3	ethyl acetate	Soxhlet apparatus	6.706	3.619	0.654
4	methanol	Soxhlet apparatus	5.486	3.440	0.485
5	acetone	Soxhlet apparatus	5.134	3.449	0.514
6	toluene	Orbital shaker	6.858	3.185	0.090
7	diethyl ether	Orbital shaker	11.886	5.724	0.713
8	hexane	Orbital shaker	7.291	4.285	0.005
9	ethyl acetate	Orbital shaker	5.929	3.086	0.393
10	methanol	Orbital shaker	5.222	2.981	0.405
11	acetone	Orbital shaker	4.632	2.029	0.446
12	ethanol	Orbital shaker	5.529	3.070	0.396
13	methanol-water	Orbital shaker	2.793	0.101	0.096
14	ethanol-water	Orbital shaker	4.916	0.740	0.298
15	acetone-water	Orbital shaker	3.573	0.250	0.303
16	toluene	UAE	6.732	3.226	0.082
17	diethyl ether	UAE	14.506	6.816	0.898
18	hexane	UAE	9.998	5.103	0.010
19	ethyl acetate	UAE	5.878	3.169	0.402
20	methanol	UAE	5.667	3.225	0.427
21	acetone	UAE	5.817	3.330	0.476
22	ethanol	UAE	5.824	3.317	0.437
23	methanol-water	UAE	1.151	0.064	0.018
24	ethanol-water	UAE	4.514	1.250	0.270
25	acetone-water	UAE	3.477	0.351	0.257
26	ChCl-Glu (+25 % water)	Orbital shaker	0.034	0.000	0.020
27	ChCl-Fru (+25 % water)	Orbital shaker	0.047	0.011	0.023
28	ChCl-LA (+25 % water)	Orbital shaker	0.404	0.069	0.393
29	ChCl-TA (+25 % water)	Orbital shaker	0.176	0.031	0.068
30	ChCl-Gly (+25 % water)	Orbital shaker	0.126	0.016	0.044
31	ChCl-EG (+25 % water)	Orbital shaker	0.448	0.031	0.273
32	ChCl-U (+25 % water)	Orbital shaker	0.164	0.000	0.046
33	ChCl-DMU (+25 % water)	Orbital shaker	3.242	0.347	0.733
34	ChCl-Phe (+25 % water)	Orbital shaker	13.043	5.666	0.890
35	ChCl-BSA (+25 % water)	Orbital shaker	0.339	0.000	0.033
36	ChCl-Glu (+25 % water)	UAE	0.053	0.044	0.016
37	ChCl-Fru (+25 % water)	UAE	0.105	0.078	0.030
38	ChCl-LA (+25 % water)	UAE	0.408	0.125	0.349
39	ChCl-TA (+25 % water)	UAE	0.369	0.185	0.062
40	ChCl-Gly (+25 % water)	UAE	0.450	0.298	0.121

41	ChCl-EG (+25 % water)	UAE	0.494	0.046	0.300
42	ChCl-U (+25 % water)	UAE	0.213	0.031	0.053
43	ChCl-DMU (+25 % water)	UAE	3.418	0.382	0.510
44	ChCl-Phe (+25 % water)	UAE	12.446	5.199	0.827
45	ChCl-BSA (+25 % water)	UAE	0.332	0.000	0.082
46	ChCl-Glu (+25 % water)	UHE	0.626	0.395	0.068
47	ChCl-Fru (+25 % water)	UHE	0.440	0.196	0.050
48	ChCl-LA (+25 % water)	UHE	1.082	0.260	0.362
49	ChCl-TA (+25 % water)	UHE	3.446	1.694	0.149
50	ChCl-Gly (+25 % water)	UHE	1.060	0.480	0.148
51	ChCl-EG (+25 % water)	UHE	0.612	0.052	0.272
52	ChCl-U (+25 % water)	UHE	0.626	0.176	0.077
53	ChCl-DMU (+25 % water)	UHE	4.526	0.635	0.463
54	ChCl-Phe (+25 % water)	UHE	10.994	4.854	0.693
55	ChCl-BSA (+25 % water)	UHE	0.053	0.000	0.000

Table S2. Statistical differences observed in extraction of α-acids, β-acids, and xanthohumol (reported as weight % of grinded hops pellets) extracted for some chosen cases in the case of ultrasound assisted extraction (UAE). The results are shown for three repeated extractions with a given solvent together with the calculated mean value and standard deviation (expressed as mean value ± standard deviation). Experimental details are reported in the main paper.

[2] – anhydrous ChCl-Phe DES was prepared first and afterwards water was added

[1] – ChCl, phenol, and 25 % (w/w) of water were put first together, heating and stirring were applied afterwards

**solvent**	**α-acids [w/w %]**		**β-acids [w/w %]**		**xanthohumol [w/w %]**	
diethyl ether	14.1583		7.2795		0.9705	
diethyl ether	13.9730	14.14±0.16	5.9293	6.85±0.79	0.9745	0.978±0.009
diethyl ether	14.2886		7.3294		0.9879	
ChCl-Phe (+25 % water) [2]	12.4150		5.3991		0.9138	
ChCl-Phe (+25 % water) [2]	12.2031	12.38±0.16	5.3370	5.42±0.10	0.8943	0.905±0.010
ChCl-Phe (+25 % water) [2]	12.5075		5.5312		0.9069	
ChCl-Phe (+25 % water) [1]	12.8410		5.6471		0.9416	
ChCl-Phe (+25 % water) [1]	12.5755	12.1±1.0	5.4600	5.22±0.58	0.9226	0.900±0.057
ChCl-Phe (+25 % water) [1]	10.9777		4.5571		0.8356	
ChCl-DMU (+25 % water)	3.5382		0.3920		0.4790	
ChCl-DMU (+25 % water)	3.5687	3.51±0.08	0.3728	0.380±0.011	0.4866	0.472±0.020
ChCl-DMU (+25 % water)	3.4193		0.3750		0.4491	
